# COVID-19-related morbidity and mortality in people with multiple long-term conditions: a systematic review and meta-analysis of over 4 million people

**DOI:** 10.1177/01410768241261507

**Published:** 2024-10-16

**Authors:** Shukrat O Salisu-Olatunji, Yogini V Chudasama, Navjot Kaur, Zara Kayani, Babatunde A Odugbemi, Olasope Esther Bolodeoku, Shirley Akua Konnor, Elpida Vounzoulaki, Atanu Bhattacharjee, Radia Fahami, Jonathan Valabhji, Amitava Banerjee, Francesco Zaccardi, Clare L Gillies, Kamlesh Khunti

**Affiliations:** 1 Leicester Real World Evidence Unit, Diabetes Research Centre, Leicester General Hospital, University of Leicester, Leicester LE5 4PW, UK; 2 Diabetes Research Centre, Leicester General Hospital, Department of Population Health Sciences, University of Leicester, Leicester, LE1 7RH, UK; 3 Department of Community Health and Primary Health Care, Lagos State University College of Medicine (LASUCOM), Lagos, Nigeria; 4 Department of Community Health and Primary Health Care, Lagos State University Teaching Hospital (LASUTH), Ikeja, Lagos, Nigeria; 5 Population Health and Genomics, Medical School, University of Dundee, Scotland, DD1 9SY, UK; 6 NHS England and Improvement, Skipton House, London, SW1A 0AA, UK; 7 Division of Metabolism, Digestion and Reproduction, Imperial College London, London, W12 0NN, UK; 8 Institute of Health Informatics, University College London, London, WC1E 6BT, UK; *Joint senior authors.

**Keywords:** Epidemiology, infectious diseases, public health

## Abstract

**Objectives:**

To describe the direct impact of coronavirus disease 2019 (COVID-19) infection on morbidity and mortality in people with multiple long-term conditions (MLTCs).

**Design:**

A systematic review and meta-analysis including observational studies.

**Setting:**

Studies conducted between 1 January 2020 and 4 May 2023 across 51 countries were identified from five databases.

**Participants:**

A total of 4,084,469 patients with confirmed COVID-19 infection.

**Main outcome measures:**

Pooled risk ratios (RRs) for mortality, hospitalisation, severe disease, intensive care unit (ICU) admission and mechanical ventilation were estimated with random effect meta-analysis models.

**Results:**

A total of 38,356 studies were identified and 111 included. In most (74%) of the studies, MLTCs referred to having two or more long-term conditions. Others described MLTCs by high weighted indices: the Charlson Comorbidity Index in 11% and the Clinical Frailty Score in 7%. Using the National Institutes of Health quality assessment tool for observational studies, the risk of bias was judged as low and moderate in 86 and 25 studies, respectively. Having MLTCs was associated with increased mortality (RR: 2.61 [95% CI: 2.27 to 3.0]); hospitalisation (2.4 [1.92 to 2.99]); severe disease (2.61 [1.92 to 3.54]); ICU admission (1.22 [1.07 to 1.39]) and mechanical ventilation (1.83 [1.18 to 2.84]) compared with those with no MLTCs. Pooled RRs for adverse outcomes were higher in children and young people compared with all age groups. In meta-regression analyses, men were more likely to need ICU admission (*p* = 0.013) and mechanical ventilation (*p* = 0.002).

**Conclusions:**

Public health policies, clinical and preventative interventions should prioritise people with MLTCs to minimise direct adverse outcomes from COVID-19 disease.

## Introduction

A global pandemic caused by the severe acute respiratory syndrome coronavirus-2 ushered in the year 2020, resulting in extensive adverse health, economic and social impacts worldwide.^1,2^ Early in the pandemic, studies highlighted the likelihood of worsening health inequalities related to socioeconomic status, healthcare access and pre-existing health conditions.^3,4^ Thus, public health efforts sought to identify and target those at high risk to minimise their morbidity and mortality.

Research has identified important risk factors for severe coronavirus disease 2019 (COVID-19) disease and mortality. These include sociodemographic factors such as older age,^5,6^ male sex,^7^ socioeconomic deprivation,^8,9^ being from ethnic minority backgrounds^10,11^ and having pre-existing conditions.^12^ However, there is limited information on the associations between having concurrent multiple long-term conditions (MLTCs) and COVID-19 disease severity and mortality. This is a growing concern considering that about one-third of adults globally (and over 25% of England’s adult population) have two or more long-term conditions concomitantly.^13,14^

MLTCs have been defined as the concurrent existence of two or more long-term physical, mental or infectious health conditions in an individual, which require ongoing treatment and result in some limitations to daily activity and quality of life.^15,16^ Measures based on disease counts or weighted indices have been used in clinical practice and research to quantify MLTCs as appropriate for the context.^17^ For instance, count measures may be preferable to weighted indices in studies involving young adults or children as some of the common weighted indices, e.g. Charlson Comorbidity Index (CCI), Clinical Frailty Score (CFS) and Cumulative Illness Rating Scale, are designed for use in frail, older populations.^18^

To date, there is limited reporting of the health outcomes following COVID-19 infection in individuals with MLTCs. This information would be useful to guide clinical management and public health interventions targeting people with MLTCs in times of such public health crises.

This systematic review and meta-analysis therefore aimed to compare the risk for COVID-19-related morbidity and mortality in people with MLTCs with those without MLTCs and explore the associations with demographic determinants.

## Methods

### Search strategy and selection criteria

The systematic review is reported following the Preferred Reporting Items for Systematic Reviews and Meta-Analyses (PRISMA) 2020 guidelines (Supplementary Tables S1). The protocol for the review was registered on 4 April 2022 on PROSPERO, (CRD42022322567).

Database searches were conducted in Medline, Scopus, CINAHL, Cochrane Library and the World Health Organization (WHO) COVID-19 Global literature on coronavirus disease for potentially relevant literature. Free text and medical subheading terms were included in the search strategy (Supplementary S2: Medline search strategy). The search was limited to English-language articles published between 1 January 2020 and 4 May 2023.

The eligibility criteria for inclusion of studies were outlined following the ‘PECOS’ framework. The ‘Population’ was patients diagnosed with COVID-19 infection (with confirmed polymerase chain reaction test or clinical diagnosis). The ‘Exposure’ was having MLTCs, as defined by count (two or more, three or more) or high weighted indices (e.g. CCI ≥ 2, CFS ≥ 5). The ‘Comparator’ was non-MLTC population (people with no long-term conditions (LTCs), single LTC or low weighted indices, e.g. CCI < 2, CFS < 5). The ‘Outcomes’ were patient deaths, hospitalisations, severe disease, intensive care unit (ICU) admission and mechanical ventilation among those with and without MLTCs. Regarding the ‘Study design’, the review included observational studies only.

The authors (SSO, NK, BAO, ZK, OEB, EV and AtB) screened the titles and abstracts of identified studies independently after excluding duplicates in the reference manager EndNote, and in Rayyan, a collaborative systematic review management software (https://www.rayyan.ai/). Conflicted decisions were resolved by discussion.

The screening of the full texts of the articles was also conducted in Rayyan by the authors (SSO, NK, BAO, ZK, RF, OEB and EV). A pair of reviewers screened a set of articles independently, and then resolved conflicted decisions by discussion. The percentage of agreement between pairs of reviewers ranged between 74% and 97% before conflicted decisions were resolved.

### Data extraction

Data including general study information, population characteristics and outcome frequency were extracted from the studies in an Excel spreadsheet. This was conducted independently by some of the authors (SSO, NK, BAO, ZK, OEB and SAK) with cross-checks for accuracy carried out among them.

Data were extracted to calculate the estimates for the risk ratios (RRs) for the outcomes of interest, comparing patients with MLTCs to those without MLTCs.

The mortality data reported in the included studies are described as COVID-19-related mortality in this review and comprise deaths from COVID-19 infection and all-cause mortality during hospitalisation in patients diagnosed with COVID-19 disease.

### Risk of bias assessment

The quality of the included studies was assessed using the National Institutes of Health (NIH) quality assessment tool for observational cohort and cross-sectional studies.^19^ This was assessed independently by the authors (SSO, NK, ZK, SAK and OEB), with discussions to reach mutual agreements where the assessments differed. The tool consists of 14 measures, and each was assigned a response of ‘Yes’, ‘No’, ‘Cannot determine’, ‘Not applicable’ or ‘Not reported’. A low risk of bias was determined where the study had a ‘Yes’ response to more than half of the 14 measures, a moderate risk of bias for ‘Yes’ to between five and seven measures, and a high risk of bias where the response was ‘Yes’ for less than five measures. These judgement parameters were defined specifically for this review by the authors as recommended by the tool developers.

### Data analysis

Statistical analyses were carried out in Stata 17.0 by three authors (SSO, CG and YC). The estimated pooled RRs and their 95% confidence intervals (CIs) for the outcomes of interest were calculated and presented in forest plots. The potential effects of the quality of the studies on the pooled risk estimates for the outcomes were explored through a sensitivity analysis. Studies reporting similar outcome measures were combined using a random effects meta-analysis model to accommodate the high between-study heterogeneity inherent in observational studies.^20^ Heterogeneity was investigated using subgroup analyses and meta-regression to explore associations between the estimated effect sizes and study-level factors such as MLTC measure used, study quality, average age and sex (proportion of men).

We assessed the likelihood of publication bias using funnel plots and Egger’s test.

## Results

**Figure 1.**
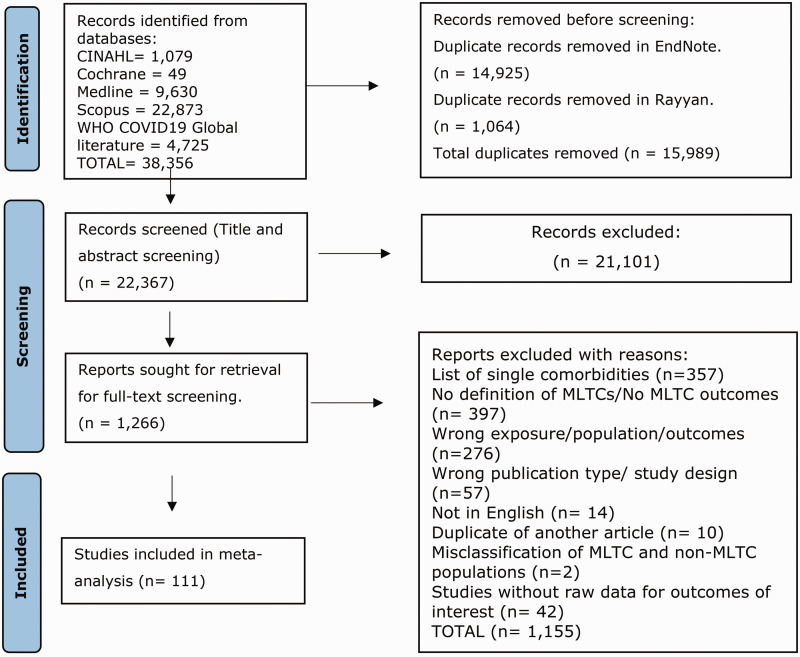
PRISMA flow diagram showing study selection.

The database searches identified 38,356 articles. Most studies excluded reported outcomes only by single comorbidities or did not define MLTCs (Figure 1). After de-duplication and screening of titles and abstracts, 1266 full-text articles were retrieved and screened for inclusion in the systematic review, of which 111 were included (Supplementary S11 – Reference list of studies).

### Study design

All 111 included studies comprised retrospective and prospective observational cohort studies and cross-sectional studies conducted between 2020 and 2023.

### Participants

A total of 4,084,469 patients were included in the studies. Of the 93 studies that reported patients’ average ages, 11 were studies involving 723,402 children and young people (under the age of 23 years).

According to The World Bank ranking of countries by income,^21^ most (55%) of the studies were conducted in high-income countries. Study locations included 51 countries across Europe, America, Asia, Africa, Australasia and the Middle East.

MLTCs were described by count as having two or more conditions or as three or more conditions in 82 and 9 studies, respectively. MLTCs were described by weighted scores such as the CCI in 12 studies and the CFS in 8 studies. The average duration of the included studies was seven months (Table 1).

**Table 1.** Summary of study characteristics.

	Study ID – first author year	Study design	Study country	Country income category	Study duration (months)	Total population	MLTC measure
1	AbouGalala 2023	Cross-sectional	Qatar	High	6	92,426	2 or more
2	Abraha 2021	Retrospective cohort	Ethiopia	Low	5	2617	2 or more
3	Accordino 2023	Retrospective cohort	Italy	High	25	300	2 or more
4	Adejumo 2022	Cross-sectional	Nigeria	Lower middle	7	2858	2 or more
5	Agrawal 2022	Retrospective cohort	Scotland	High	7	4684	2 or more
6	Akcay 2022	Retrospective cohort	Turkey	Upper middle	14	335	2 or more
7	Akerele 2021	Retrospective cohort	Nigeria	Lower middle	4	201	2 or more
8	Akhavizadegan 2021	Retrospective cohort	Iran	Lower middle	5	522	2 or more
9	Akhtar 2021	Retrospective cohort	Pakistan	Lower middle	7	1812	2 or more
10	Al Ani 2022	Cross-sectional	Iraq	Upper middle	8	34,687	2 or more
11	Al Bastaki 2022	Retrospective cohort	United Arab Emirates	High	2	5685	2 or more
12	Al Kuwari 2020	Retrospective cohort	Qatar	High	3	101	2 or more
13	Allameh 2020	Retrospective cohort	Iran	Lower middle	1	905	2 or more
14	Almalki 2020	Cross-sectional	Saudi Arabia	High	3	458	2 or more
15	Almarashda 2022	Cross-sectional	United Arab Emirates	High	13	585	3 or more
16	Amit 2020	Retrospective cohort	Israel	High	2	156	2 or more
17	Anderegg 2022	Retrospective cohort	Switzerland	High	12	22,648	2 or more
18	Andrew 2022	Retrospective cohort	Canada	High	3	2031	CFS ≥ 5
19	Arnau-Barres 2021	Retrospective cohort	Spain	High	1	405	CCI ≥ 2
20	Asem 2021	Retrospective cohort	Egypt	Lower middle	3	8162	2 or more
21	Ayed 2021	Retrospective cohort	Kuwait	High	2	103	2 or more
22	Badin 2023	Retrospective cohort	Brazil	Upper middle	17	530	2 or more
23	Bahl 2020	Retrospective cohort	USA	High	1	1461	2 or more
24	Bailly 2022	Retrospective cohort	France	High	7	1,34,209	CCI ≥ 2
25	Beaumont 2022	Retrospective cohort	France	High	1	399	2 or more
26	Benderra 2021	Retrospective cohort	France	High	3	1148	2 or more
27	Beurnier 2020	Prospective cohort	France	High	1	112	2 or more
28	Blayney 2022	Retrospective cohort	Scotland	High	18	2236	2 or more
29	Brandao-Neto 2021	Prospective cohort	Brazil	Upper middle	2	506	2 or more
30	Bucholc 2022	Retrospective cohort	United Kingdom	High	NA	6036	2 or more
31	Buckner 2020	Retrospective cohort	USA	High	2	105	3 or more
32	Bustoz-Vasquez 2021	Retrospective cohort	Mexico	Upper middle	2	16,380	2 or more
33	Buttenschon 2022	Retrospective cohort	Denmark	High	9	311	2 or more
34	Camacho Moll 2023	Retrospective cohort	Mexico	Upper middle	17	25,722	2 or more
35	Campbell 2022	Retrospective cohort	USA	High	1	1877	2 or more
36	Cardinal-Fernandez 2021	Retrospective cohort	Spain	High	1	1331	2 or more
37	Cardoso 2022	Cross-sectional	Brazil	Upper middle	18	6,71,593	2 or more
38	Catalano 2023	Retrospective cohort	Italy	High	3	12,793	CCI ≥ 2
39	Colnago 2022	Retrospective cohort	Brazil	Upper middle	2	50,896	3 or more
40	Covino 2021	Prospective cohort	Italy	High	12	729	CCI ≥ 2
41	d’Arminio-Monforte 2020	Prospective cohort	Italy	High	3	539	2 or more
42	d’Etienne 2022	Retrospective cohort	USA	High	10	7023	2 or more
43	Dandachi 2021	Retrospective cohort	USA	High	3	286	2 or more
44	Dantas 2022	Retrospective cohort	Brazil	Upper middle	15	783	2 or more
45	de Oliveira Lima 2022	Retrospective cohort	Brazil	Upper middle	12	38,937	3 or more
46	Di Fusco 2022	Retrospective cohort	USA	High	6	4573	3 or more
47	Diaz Valez 2021	Retrospective cohort	Peru	Upper middle	2	493	2 or more
48	Dragano 2022	Retrospective cohort	Germany	High	18	6,88,705	2 or more
49	Elavarasi 2022	Retrospective cohort	India	Lower middle	2	978	2 or more
50	Fagard 2022	Retrospective cohort	Belgium	High	2	105	CFS ≥ 5
51	Farrar 2022	Prospective cohort	Canada	High	14	544	2 or more
52	Funk 2022	Retrospective cohort	Czechia, Finland, Ireland, Italy, Luxembourg, Malta, Norway, Poland and Slovakia	High	6	7,63,674	2 or more
53	Galang 2021	Longitudinal surveillance	USA	High	12	7950	2 or more
54	Gani 2022	Retrospective cohort	Malaysia	Upper middle	3	228	2 or more
55	Guan 2020	Retrospective cohort	China	Upper middle	2	1590	2 or more
56	Hardelid 2022	Retrospective cohort	Scotland	High	10	19,014	2 or more
57	Hendler 2021	Retrospective cohort	Brazil	Upper middle	10	288	2 or more
58	Henkens 2022	Retrospective cohort	Netherlands	High	6	4806	3 or more
59	Hesni 2022	Retrospective cohort	Iran	Lower middle	12	27,256	2 or more
60	Ho 2020	Retrospective cohort	UK	High	3	4,70,034	3 or more
61	Houvessou 2022	Retrospective cohort	Brazil	Upper middle	11	72,647	2 or more
62	Impouma 2022	Retrospective cohort	WHO African Region	Low	7	46,870	2 or more
63	Islam 2021	Retrospective cohort	Bangladesh	Lower middle	3	174	2 or more
64	Ismail 2021	Cross-sectional	Qatar	High	3	3515	2 or more
65	Izzy 2020	Retrospective cohort	USA	High	3	5190	3 or more
66	Jachymek 2022	Retrospective cohort	Poland	High	5	201	CFS ≥ 5
67	Jesmani 2023	Retrospective cohort	Iran	Lower middle	1	665	2 or more
68	Kammar-Garcia 2020	Retrospective cohort	Mexico	Upper middle	4	13,842	2 or more
69	Khalifa 2022	Retrospective cohort	Libya	Upper middle	8	94	2 or more
70	Kofahi 2022	Cross-sectional	Jordan	Lower middle	4	2148	3 or more
71	Kokoszka-Bargiel 2022	Retrospective cohort	Poland	High	12	113	CFS ≥ 5
72	Koyyada 2022	Retrospective cohort	India	Lower middle	6	369	2 or more
73	Kruger 2022	Retrospective cohort	South Africa	Upper middle	3	2508	2 or more
74	Kuhn 2022	Retrospective cohort	USA	High	12	1,24,925	2 or more
75	Kumar 2021	Prospective cohort	India	Lower middle	5	109	2 or more
76	Lam 2022	Retrospective cohort	USA	High	15	6865	CCI ≥ 2
77	Larsson 2020	Retrospective cohort	Sweden	High	2	260	2 or more
78	Levy 2022	Retrospective cohort	Israel	High	8	849	CCI ≥ 2
79	Lota-Salvado 2023	Cross-sectional	Philippines	Lower middle	24	115	2 or more
80	Marengoni 2021	Retrospective cohort	Italy	High	1	165	CFS ≥ 5
81	Marin-Gomez 2022	Retrospective cohort	Spain	High	2	37,110	2 or more
82	Marti-Pastor 2023	Retrospective cohort	Spain	High	14	785	CFS ≥ 5
83	Mertens 2022	Cross-sectional	Belgium	High	16	44,550	2 or more
84	Mi 2020	Retrospective cohort	China	Upper middle	2	189	2 or more
85	Mohammadifard 2022	Retrospective cohort	Iran	Lower middle	4	4356	2 or more
86	Monari 2022	Prospective cohort	Italy	High	14	329	CCI ≥ 2
87	Ngere 2022	Cross-sectional	Kenya	Lower middle	5	2796	2 or more
88	Oliveira 2023	Retrospective cohort	Brazil	Upper middle	3	7442	2 or more
89	Pinzon 2023	Retrospective cohort	Indonesia	Upper middle	NR	333	2 or more
90	Polverino 2020	Prospective cohort	Italy	High	1	3179	2 or more
91	Quenzer 2022	Retrospective cohort	US–Mexico border	Upper middle	2	156	2 or more
92	Rainer 2022	Retrospective cohort	Austria	High	22	42,442	CCI ≥ 2
93	Rana 2023	Retrospective cohort	India	Lower middle	7	2586	2 or more
94	Rando 2023	Retrospective cohort	Italy	High	2	258	2 or more
95	Rossi 2020	Prospective cohort	Italy	High	1	2653	CCI ≥ 2
96	Semenzato 2021	Retrospective cohort	France	High	4	87,809	2 or more
97	Siddiqi 2022	Retrospective cohort	India	Lower middle	6	104	2 or more
98	Simoes 2022	Retrospective cohort	Brazil	Upper middle	15	477	2 or more
99	Siqueira 2022	Retrospective cohort	Brazil	Upper middle	19	15,105	2 or more
100	Skarbinski 2022	Retrospective cohort	United States of America	High	6	6624	CCI ≥ 2
101	Smith JP 2022	Longitudinal cohort	United States of America	High	11	6357	2 or more
102	Solanki 2022	Cross-sectional	South Africa	Upper middle	12	1,88,292	2 or more
103	Subramaniam 2022	Retrospective cohort	Australia and New Zealand	High	24	3077	CFS ≥ 5
104	Sundaram 2022	Retrospective cohort	United States of America	High	12	1,00,902	CCI ≥ 2
105	Surendra 2023	Retrospective cohort	Indonesia	Lower middle	7	6583	2 or more
106	Tam 2022	Retrospective cohort	Hong Kong	High	2	101	CFS ≥ 5
107	Toofan 2021	Retrospective cohort	Iran	Lower middle	7	2597	2 or more
108	Traiber 2022	Retrospective cohort	Brazil	Upper Middle	12	32	2 or more
109	Wiley 2022	Retrospective cohort	United States of America	High	10	7155	CCI ≥ 2
110	Zhang C 2022	Retrospective cohort	China	Upper middle	1.5	62	2 or more
111	Zhang Y 2022	Retrospective cohort	China	Upper middle	1.5	80,543	2 or more

CCI: Charlson Comorbidity Index; CFS: Clinical Frailty Scale.

### Risk of bias

The 14 criteria in the NIH tool for assessing the quality of observational studies were applied to the studies (Supplementary Table S3). A total of 86 and 25 studies were assessed as having low and moderate risk of bias, respectively (Supplementary Table S4) The risk of bias graph (Supplementary Figure S5) shows the distribution of the responses to each criterion among the studies. Most studies clearly stated the research objectives (108/111), clearly defined the study population (106/111), recruited from the same or similar populations with prespecified inclusion and exclusion criteria (104/111) and clearly defined the outcome measures (107/111).

Publication bias was likely only for the outcome of mortality. This could reflect published studies focusing on mortality as a key outcome during the pandemic and as such, studies with low or no mortality may be unpublished (Supplementary Figure S6).

### Primary study outcomes

**Figure 2.**
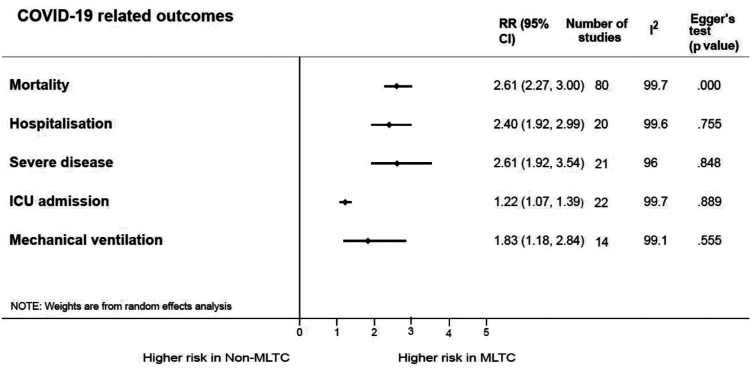
Pooled relative risks for COVID-19-related outcomes in MLTC and non-MLTC populations.

**Figure 3.**
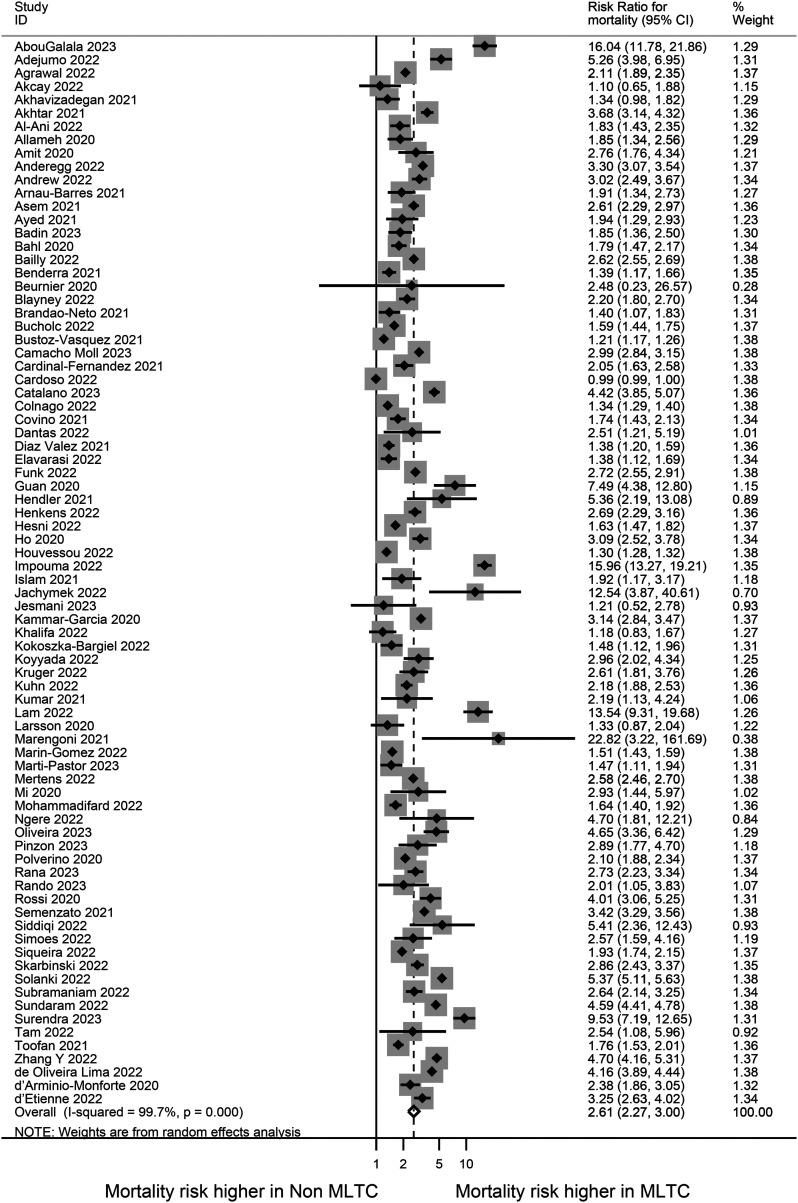
Forest plot showing the meta-analysis for risk ratio for mortality in MLTC vs non-MLTC populations.

The summary of the pooled risk estimates for the outcomes of interest is presented in Figure 2. Mortality was the most reported outcome (reported in 80 studies), as presented in a forest plot showing the effect estimates and 95% CIs (Figure 3). Patients with MLTCs had more than twice the risk for mortality compared with patients without MLTCs [RR: 2.61 (95% CI: 2.27 to 3.0)]. Four studies involving only children and young people reported mortality and the risk was higher in those with MLTC compared with those without MLTCs [2.84 (1.41 to 5.70)] (Supplementary Figure S7a).

Hospitalisation was reported in 20 studies that involved patients from various settings. Those with MLTCs had a greater than two-fold risk for hospitalisation compared with those without MLTCs [2.40 (1.92 to 2.99)] (Supplementary Figure S7b). In the studies involving only children and young people, the RR for hospitalisation among those with MLTCs compared with those without MLTCs was much higher [3.53 (1.07 to 11.66)] (Supplementary Figure S7c).

A total of 21 studies compared the severity of COVID-19 disease in patients with MLTCs with those without MLTCs and found more than double the risk in those with MLTCs [2.61 (1.92 to 3.54)] (Supplementary Figure S7d). Most of these studies defined severity based on presenting symptoms, the types of therapy and treatment support required as defined by WHO or other guidelines.^22,23^ Some studies alternatively described disease severity as a composite endpoint of outcomes such as ICU admission, IMV or death.^24^

Among the patients in the 22 studies that reported ICU admission rates between patients with MLTCs and those without, the pooled risk estimate was 22% higher in patients with MLTCs, indicating an increased need for ICU admission [1.22 (1.07 to 1.39)] (Supplementary Figure S7e). In studies reporting age, those involving only children and young people reported an even higher RR among those with MLTCs [2.90 (1.96 to 4.29)] (Supplementary Figure S7f).

Patients with MLTCs in the 14 studies that reported the need for mechanical ventilation had an 83% higher likelihood for mechanical ventilation compared with those without MLTCs [1.83 (1.18 to 2.84)] (Supplementary Figure S7g). The studies involving only children and young people reported higher RRs between those with MLTCs and those without MLTCs needing mechanical ventilation [4.29 (1.14 to 16.18)] (Supplementary Figure S7h).

### Sensitivity and subgroup analyses

The likelihood of differences in pooled risk estimates due to differences in the quality of the studies was explored through a sensitivity analysis. This was based on the risk of bias assessments as a measure of study quality (studies with a low risk of bias are described as having good quality). The pooled risk estimate for mortality in studies of good quality [2.63 (2.19 to 3.14)] was similar for studies with fair quality (moderate risk of bias) [2.6 (1.7 to 3.99)] (Supplementary Figure S8a). This was the same for hospitalisation and severe disease with no statistically significant difference in the risk estimates between studies with good and fair quality (Supplementary Figure S8b, c).

With ICU admission and mechanical ventilation, however, the studies with moderate risk of bias had significantly higher pooled risk estimates but these studies were few (two and one, respectively), thus limiting the inclusion in the interpretation of a meta-analysis (Supplementary Figures S8d, S8e and S9d, S9e).

Statistical heterogeneity was high (>75%) across all the studies included in the meta-analyses. In the exploration of between-study heterogeneity, sub-group analyses found no statistically significant difference in the pooled RRs for mortality between lower middle [2.5 (1.97 to 3.17)], upper middle [2.3 (1.88 to 2.81)] and high-income [2.66 (2.35 to 3.0)] countries, but the risk estimate was significantly higher in the low-income country [15.96 (13.27 to 19.21)], although this was only reported in one study (Table 2).

**Table 2.** Summary of subgroup analysis for the risk ratio for mortality.

Variable	Pooled risk estimates for MLTC vs no MLTC (95% confidence intervals)	Number of studies included in the analysis	*I*^2^ (%)
Country income group			
Low income	15.96 (13.27 to 19.21)	1^a^	.
Lower-middle income	2.50 (1.97 to 3.17)	17	94.4
Upper-middle income	2.30 (1.88 to 2.81)	24	99.8
High income	2.66 (2.35 to 3.0)	38	98.1
MLTC measure			
2 or more	2.47 (2.13 to 2.86)	61	99.7
3 or more	2.61 (1.28 to 5.31)	4	99.7
CFS ≥ 5	2.56 (1.77 to 3.71)	7	85.7
CCI ≥ 2	3.54 (2.66 to 4.7)	8	98.9
Study duration (months)			
≤7	2.70 (2.36 to 3.1)	54	98.7
>7	2.41 (1.87 to 3.11)	24	99.9
Study quality (risk of bias)			
Low risk of bias	2.63 (2.19 to 3.14)	65	99.8
Moderate risk of bias	2.60 (1.7 to 3.99)	15	98.3

a Not included in the meta-analysis.

The MLTC measure used was also explored with a similar two-fold higher risk for mortality in patients with MLTCs compared with those without MLTCs in studies that defined MLTC as two or more conditions [2.47 (2.13 to 2.86), three or more conditions [2.61 (1.28 to 5.31)], and CFS ≥ 5 [2.56 (1.77 to 3.71)]. The studies that reported CCI (CCI ≥ 2) as the MLTC measure had a slightly higher risk in patients with MLTCs compared with those without MLTCs [3.54 (2.66 to 4.7)] (Supplementary Table S9a).

Summary-level data for sociodemographic factors such as deprivation status and ethnicity were inconsistently reported in the studies. In total, 17 studies reported patients’ ethnicity with various descriptors such as race, skin colour and nationality but there was no report by MLTC status to allow for exploring associations between ethnicity and outcomes. While some used categories such as White, Black, Asian and others, other studies included Hispanic and non-Hispanic subgroups, and one reported a dichotomous nationality variable – Qatari and non-Qatari. Regarding socioeconomic status, various measures, such as deprivation index, income, household employment status, composite socioeconomic index, education and type of insurance cover, were also inconsistently reported in a few studies.

For the other outcomes, subgroup analysis based on study-level characteristics showed there was no statistically significant difference in the pooled RRs for the outcomes between the subgroups concerning country income group, MLTC measure used, study duration or study quality (Supplementary Table S9).

### Meta-regression analyses

Meta-regression analyses found that the study-level characteristics of average age and sex distribution of study participants (proportion of men) were not significantly associated with the estimated RRs for mortality, hospitalisation and severe disease in patients who had MLTCs and those who did not. However, there was a positive relationship between male sex and a higher risk for ICU admission (*p* = 0.013) and mechanical ventilation (*p* = 0.002) (Supplementary Table S10 and Figure S11).

## Discussion

### Principal findings

This review demonstrated a significantly higher risk of adverse outcomes following COVID-19 infection among people living with MLTCs compared with those without MLTCs. Study estimates of risk were not found to be associated with study-level determinants such as age, the MLTC measure used, the country’s income group, study quality or duration. However, the inadequate and inconsistent reporting of summary-level sociodemographic data precluded exploration of the relationships between the outcomes of interest and these determinants for a more robust review.

### Study strengths

To our knowledge, this is the largest cohort of patients with COVID-19 included in a systematic review investigating outcomes in people with MLTCs. This review assessed COVID-19-related outcomes in people of different age groups (children, young people and adults). We included different definitions of MLTCs by count and weighted indices for a more robust review. We also explored the effects of some determinants such as study characteristics, on the risk estimates for the outcomes in the MLTC and non-MLTC populations. Additionally, the inclusion of a large cohort of over 4 million study participants across 51 countries provides a more global overview of COVID-19 and MLTCs.

### Comparison with previous studies

Previous studies have indicated that age and sex may modify the association between the presence or absence of pre-existing comorbidities and adverse COVID-19 outcomes. This systematic review reporting study-level measures of age and sex, however, found that the risk estimates for mortality, hospitalisation and having severe disease were not likely influenced by age and sex distribution across the population. However, being a man was associated with a higher risk of requiring ICU admission and mechanical ventilation.

Although previous systematic reviews assessing comorbidities and the risk for severe COVID-19 disease and mortality have examined these associations concerning single conditions,^25,26^ this review found a similarly higher risk for severe disease and mortality in the presence of multiple comorbidities.

Severe disease was more likely in individuals with MLTCs in previous studies; however, one study among children contradictorily found that severe COVID-19 disease was more common in children who did not have multiple underlying conditions,^24^ whereas other studies among children more plausibly reported a six-fold increase,^22,27^ and twice the risk^28^ for severe COVID-19 disease among children with multiple underlying conditions. Similarly, this review found higher RRs for the outcomes of interest among those with MLTCs in studies among children and young people compared with studies involving patients of all ages. This is an interesting finding that may suggest that increased vulnerabilities could be associated more with extremes of age (the youngest and oldest) rather than increasing age.

In contrast to this review, previous systematic reviews have included a smaller number of participants or shorter study periods. For instance, Singh et al. included 18 studies up to April 2020 with 14,558 participants.^25^ Yang et al. had 16 studies up to October 2020 and included 4324 patients.^29^ Saragih et al. had 22 studies among 924,520 frail and non-frail elderly participants studied up until March 2021.^30^ The present systematic review therefore potentially provides an updated picture of the impact of COVID-19 infection on outcomes among people with MLTCs.

### Future research

The variation in MLTC measures and outcome data reported across studies undermined the strength of the evidence that could be derived from the synthesis of observational studies on the outcomes of COVID-19 infection in this vulnerable population. Efforts to improve the homogeneity of outcome measures reported in similar studies would be a welcome development and should be considered in future MLTC research. The expert consensus recommendation to include a minimum list of conditions to define MLTCs as having two or more of the listed conditions is therefore laudable.^15^ Disease counts present a more inclusive measure for different age groups and frailty levels to enhance comparability and synthesis among studies. As was seen in this review, MLTC was measured by count as two or more LTCs in most of the studies and all the studies among children.

## Conclusion

The evidence from this systematic review supports the need for public health interventions for people with MLTCs to minimise their risk of adverse health outcomes. It is hoped that health policies will consider people with MLTCs for timely preventative interventions, especially in times of public health emergencies such as the COVID-19 pandemic.

## Supplemental Material

sj-pdf-1-jrs-10.1177_01410768241261507 - Supplemental material for COVID-19-related morbidity and mortality in people with multiple long-term conditions: a systematic review and meta-analysis of over 4 million people Supplemental material, sj-pdf-1-jrs-10.1177_01410768241261507 for COVID-19-related morbidity and mortality in people with multiple long-term conditions: a systematic review and meta-analysis of over 4 million people by Shukrat O Salisu-Olatunji, Yogini V Chudasama, Navjot Kaur, Zara Kayani, Babatunde A Odugbemi, Olasope Esther Bolodeoku, Shirley Akua Konnor, Elpida Vounzoulaki, Atanu Bhattacharjee, Radia Fahami, Jonathan Valabhji, Amitava Banerjee, Francesco Zaccardi, Clare L Gillies and Kamlesh Khunti in Journal of the Royal Society of Medicine

## Declarations

### Guarantor

CLG.

### Provenance

Not commissioned; peer reviewed by Julie Morris.
