# Supplementary material for: COVID-19-related morbidity and mortality in people with multiple long-term conditions: a systematic review and meta-analysis of over 4 million people
Source: J R Soc Med. 2024 Oct 16:01410768241261507. Online ahead of print. doi: 10.1177/01410768241261507 (PMC11561988; doi:10.1177/01410768241261507)
Supplement: sj-pdf-1-jrs-10.1177_01410768241261507 - Supplemental material for COVID-19-related morbidity and mortality in people with multiple long-term conditions: a systematic review and meta-analysis of over 4 million people [file sj-pdf-1-jrs-10.1177_01410768241261507.pdf]

TABLE OF CONTENTS

Contents

S1: PRISMA CHECKLIST 2

S2: MEDLINE SEARCH STRATEGY 5

S3: NIH CRITERIA 7

S4: QUALITY ASSESSMENT FOR 111 INCLUDED STUDIES 8

S5: SUMMARY OF RESPONSES FOR RISK OF BIAS ASSESSSMENT WITH NIH TOOL 15

S6: FUNNEL PLOTS FOR ASSESSMENT OF PUBLICATION BIAS 16

S7: FOREST PLOTS FOR COVID-19-RELATED OUTCOMES 18

S8: FOREST PLOTS FOR SENSITIVITY ANALYSES (STUDY QUALITY) 26

S9: Tables showing summary of sub-group analysis for pooled risk estimates for COVID-19-related outcomes 31

S10: Results of meta-regression models assessing the effects of continuous variables on the risk ratio for outcomes 34

S11: Bubble plots for meta-regression for proportion of males and ICU admission, Mechanical ventilation 35

S12: LIST OF REFERENCES FOR 111 STUDIES INCLUDED IN THE SYSTEMATIC REVIEW 36

S1: PRISMA CHECKLIST

Section and Topic	Item #	Checklist item	Location where item is reported
**TITLE**			
Title	1	Identify the report as a systematic review.	Page 1
**ABSTRACT**			
Abstract	2	See the PRISMA 2020 for Abstracts checklist.	Page 4
**INTRODUCTION**			
Rationale	3	Describe the rationale for the review in the context of existing knowledge.	Pages 5
Objectives	4	Provide an explicit statement of the objective(s) or question(s) the review addresses.	Page 5
**METHODS**			
Eligibility criteria	5	Specify the inclusion and exclusion criteria for the review and how studies were grouped for the syntheses.	Pages 6
Information sources	6	Specify all databases, registers, websites, organisations, reference lists and other sources searched or consulted to identify studies. Specify the date when each source was last searched or consulted.	Page 6
Search strategy	7	Present the full search strategies for all databases, registers and websites, including any filters and limits used.	Page 6 and S2
Selection process	8	Specify the methods used to decide whether a study met the inclusion criteria of the review, including how many reviewers screened each record and each report retrieved, whether they worked independently, and if applicable, details of automation tools used in the process.	Page 6-7
Data collection process	9	Specify the methods used to collect data from reports, including how many reviewers collected data from each report, whether they worked independently, any processes for obtaining or confirming data from study investigators, and if applicable, details of automation tools used in the process.	Page 7
Data items	10a	List and define all outcomes for which data were sought. Specify whether all results that were compatible with each outcome domain in each study were sought (e.g. for all measures, time points, analyses), and if not, the methods used to decide which results to collect.	Page 7
	10b	List and define all other variables for which data were sought (e.g. participant and intervention characteristics, funding sources). Describe any assumptions made about any missing or unclear information.	Page 7
Study risk of bias assessment	11	Specify the methods used to assess risk of bias in the included studies, including details of the tool(s) used, how many reviewers assessed each study and whether they worked independently, and if applicable, details of automation tools used in the process.	Page 7
Effect measures	12	Specify for each outcome the effect measure(s) (e.g. risk ratio, mean difference) used in the synthesis or presentation of results.	Page 6-7
Synthesis methods	13a	Describe the processes used to decide which studies were eligible for each synthesis (e.g. tabulating the study intervention characteristics and comparing against the planned groups for each synthesis (item #5)).	Page 7
	13b	Describe any methods required to prepare the data for presentation or synthesis, such as handling of missing summary statistics, or data conversions.	N/A

Section and Topic	Item #	Checklist item	Location where item is reported
	13c	Describe any methods used to tabulate or visually display results of individual studies and syntheses.	Page 7
	13d	Describe any methods used to synthesize results and provide a rationale for the choice(s). If meta-analysis was performed, describe the model(s), method(s) to identify the presence and extent of statistical heterogeneity, and software package(s) used.	Page 7
	13e	Describe any methods used to explore possible causes of heterogeneity among study results (e.g. subgroup analysis, meta-regression).	Page 7
	13f	Describe any sensitivity analyses conducted to assess robustness of the synthesized results.	Page 7
Reporting bias assessment	14	Describe any methods used to assess risk of bias due to missing results in a synthesis (arising from reporting biases).	Page 7
Certainty assessment	15	Describe any methods used to assess certainty (or confidence) in the body of evidence for an outcome.	Page 7
**RESULTS**			
Study selection	16a	Describe the results of the search and selection process, from the number of records identified in the search to the number of studies included in the review, ideally using a flow diagram.	Figure 1
	16b	Cite studies that might appear to meet the inclusion criteria, but which were excluded, and explain why they were excluded.	
Study characteristics	17	Cite each included study and present its characteristics.	Table 1 Suppl S11
Risk of bias in studies	18	Present assessments of risk of bias for each included study.	Suppl. S4
Results of individual studies	19	For all outcomes, present, for each study: (a) summary statistics for each group (where appropriate) and (b) an effect estimate and its precision (e.g. confidence/credible interval), ideally using structured tables or plots.	Figure 3
Results of syntheses	20a	For each synthesis, briefly summarise the characteristics and risk of bias among contributing studies.	
	20b	Present results of all statistical syntheses conducted. If meta-analysis was done, present for each the summary estimate and its precision (e.g. confidence/credible interval) and measures of statistical heterogeneity. If comparing groups, describe the direction of the effect.	Figure 3
	20c	Present results of all investigations of possible causes of heterogeneity among study results.	N/R
	20d	Present results of all sensitivity analyses conducted to assess the robustness of the synthesized results.	Suppl 8-10
Reporting biases	21	Present assessments of risk of bias due to missing results (arising from reporting biases) for each synthesis assessed.	N/R
Certainty of evidence	22	Present assessments of certainty (or confidence) in the body of evidence for each outcome assessed.	N/R
**DISCUSSION**			
Discussion	23a	Provide a general interpretation of the results in the context of other evidence.	Page 11-13
	23b	Discuss any limitations of the evidence included in the review.	Page 12
	23c	Discuss any limitations of the review processes used.	Page 6

Section and Topic	Item #	Checklist item	Location where item is reported
	23d	Discuss implications of the results for practice, policy, and future research.	Page 13
**OTHER INFORMATION**			
Registration and protocol	24a	Provide registration information for the review, including register name and registration number, or state that the review was not registered.	Page 6
	24b	Indicate where the review protocol can be accessed, or state that a protocol was not prepared.	Page 6
	24c	Describe and explain any amendments to information provided at registration or in the protocol.	N/A
Support	25	Describe sources of financial or non-financial support for the review, and the role of the funders or sponsors in the review.	Page 2
Competing interests	26	Declare any competing interests of review authors.	Page 2
Availability of data, code and other materials	27	Report which of the following are publicly available and where they can be found: template data collection forms; data extracted from included studies; data used for all analyses; analytic code; any other materials used in the review.	N/A

-

S2: MEDLINE SEARCH STRATEGY

Ovid MEDLINE(R) ALL <1946 to April 11, 2022>

1 exp comorbidity/ or exp multimorbidity/ 123401

2 (co morbid* or co-morbid*).mp. [mp=title, abstract, original title, name of substance word, subject heading word, floating sub-heading word, keyword heading word, organism supplementary concept word, protocol supplementary concept word, rare disease supplementary concept word, unique identifier, synonyms] 30829

3 (multi-morbid* or multimorbid*).mp. [mp=title, abstract, original title, name of substance word, subject heading word, floating sub-heading word, keyword heading word, organism supplementary concept word, protocol supplementary concept word, rare disease supplementary concept word, unique identifier, synonyms] 8416

4 "multiple long-term conditions".mp. [mp=title, abstract, original title, name of substance word, subject heading word, floating sub-heading word, keyword heading word, organism supplementary concept word, protocol supplementary concept word, rare disease supplementary concept word, unique identifier, synonyms] 73

5 exp chronic disease/ or exp multiple chronic conditions/ or noncommunicable diseases/ 592164

6 1 or 2 or 3 or 4 or 5 728665

7 exp covid-19/ or exp severe acute respiratory syndrome/ 156918

8 (covid 19 or covid-19 or covid 2019 or covid).mp. [mp=title, abstract, original title, name of substance word, subject heading word, floating sub-heading word, keyword heading word, organism supplementary concept word, protocol supplementary concept word, rare disease supplementary concept word, unique identifier, synonyms] 236177

9 exp COVID-19/ or exp Severe Acute Respiratory Syndrome/ or exp Betacoronavirus/ or exp SARS-CoV-2/ or exp Coronavirus Infections/ 168060

10 novel coronavirus.mp. [mp=title, abstract, original title, name of substance word, subject heading word, floating sub-heading word, keyword heading word, organism supplementary concept word, protocol supplementary concept word, rare disease supplementary concept word, unique identifier, synonyms] 11262

11 exp Coronavirus/ or exp Middle East Respiratory Syndrome Coronavirus/ 133633

12 severe acute respiratory syndrome coronavirus 2.mp. [mp=title, abstract, original title, name of substance word, subject heading word, floating sub-heading word, keyword heading word, organism supplementary concept word, protocol supplementary concept word, rare disease supplementary concept word, unique identifier, synonyms] 23773

13 7 or 8 or 9 or 10 or 11 or 12 254532

14 6 and 13 6935

15 limit 14 to (english language and yr="2020 -Current") **6616**
16 limit 15 to (english language and humans and yr="2020 -Current") **6169**

S3: NIH CRITERIA

NIH Quality assessment tool for observational cohort and cross-sectional studies - Risk of bias assessment

A Was the research question or objective in this paper clearly stated?
B Was the study population clearly specified and defined?
C Was the participation rate of eligible persons at least 50%?
D Were all the subjects selected or recruited from the same or similar populations (including the same time period)? Were inclusion and exclusion criteria for being in the study prespecified and applied uniformly to all participants?
E Was a sample size justification, power description, or variance and effect estimates provided?
F For the analyses in this paper, were the exposure(s) of interest measured prior to the outcome(s) being measured?
G Was the timeframe sufficient so that one could reasonably expect to see an association between exposure and outcomes if it existed?
H For exposures that can vary in amount or level, did the study examine different levels of the exposure as related to the outcome (e.g., categories of exposure, or exposure measured as a continuous variable)?
I Were the exposure measures (independent variables) clearly defined, valid, reliable, and implemented consistently across all study participants?
J Was the exposure(s) assessed more than once over time?
K Were the outcome measures (dependent variables) clearly defined, valid, reliable, and implemented consistently across all study participants?
L Were the outcome assessors blinded to the exposure status of participants?
M Was loss to follow-up after baseline 20% or less?
N* Were key potential confounding variables measured and adjusted statistically for their impact on the relationship between exposure(s) and outcome(s)?

Key to responses

Response	Meaning
Y	Yes
N	No
Others	Cannot determine, Not reported, Not applicable

S4: QUALITY ASSESSMENT FOR 111 INCLUDED STUDIES

Study 1st Author and year	A	B	C	D	E	F	G	H	I	J	K	L	M	N	Total Yes	Overall risk of bias
AbouGalala 2023	Y	Y	Y	Y	N	Y	N	Y	Y	N	Y	CD	Y	Y	10	Low
Abraha, 2021	Y	Y	Y	Y	N	Y	N	N	Y	N	Y	NA	Y	Y	9	Low
Accordino 2023	Y	Y	Y	Y	N	CD	N	Y	Y	N	N	NA	Y	N	7	Moderate
Adejumo 2022	Y	Y	Y	Y	N	Y	N	Y	Y	N	Y	NA	Y	N	9	Low
Agrawal 2022	Y	Y	Y	Y	N	CD	N	Y	Y	N	Y	NA	Y	Y	9	Low
Akcay 2022	Y	Y	Y	Y	N	Y	CD	N	Y	N	N	NA	Y	N	7	Moderate
Akerele 2021	Y	Y	Y	Y	N	Y	N	Y	Y	N	Y	NA	Y	N	9	Low
Akhavizadegan 2021	Y	Y	Y	Y	N	Y	N	Y	Y	N	Y	NA	Y	N	9	Low
Akhtar 2021	Y	Y	Y	Y	N	N	NA	Y	Y	N	Y	NA	Y	NR	8	Low
Al Ani 2022	Y	Y	CD	Y	N	N	CD	Y	Y	N	Y	NA	CD	NR	6	Moderate
Al Bastaki 2022	Y	Y	Y	Y	N	Y	CD	Y	Y	N	Y	NR	Y	Y	10	Low
Al Kuwari 2020	Y	Y	Y	Y	N	N	N	Y	Y	N	Y	NA	Y	N	8	Low
Allameh 2020	Y	Y	Y	Y	Y	Y	CD	Y	Y	N	Y	NA	Y	Y	11	Low
Almalki 2020	Y	Y	NR	Y	N	N	CD	Y	Y	N	Y	NA	CD	N	6	Moderate
Almarashda 2022	Y	Y	Y	Y	Y	Y	CD	Y	Y	N	Y	NR	Y	NR	10	Low

Amit 2020	Y	Y	CD	Y	N	N	CD	Y	Y	N	Y	NA	Y	N	7	Moderate
Anderegg 2022	Y	Y	Y	Y	N	Y	CD	Y	Y	N	Y	NR	Y	NR	9	Low
Andrew 2022	Y	Y	CD	Y	N	Y	NA	Y	Y	N	Y	NR	Y	NR	8	Low
Arnau-Barres 2021	Y	Y	Y	Y	N	N	CD	Y	Y	N	Y	NA	Y	NR	8	Low
Asem 2021	Y	Y	Y	Y	N	N	CD	Y	Y	N	Y	NA	Y	N	8	Low
Ayed 2021	Y	Y	Y	Y	N	N	N	Y	Y	N	Y	NA	Y	N	8	Low
Badin 2023	Y	N	CD	Y	N	Y	CD	Y	Y	N	Y	NR	CD	N	6	Moderate
Bahl 2020	Y	Y	Y	Y	N	N	NA	Y	Y	N	Y	NA	Y	N	8	Low
Bailly 2022	Y	Y	Y	Y	N	Y	CD	Y	Y	N	Y	NA	Y	Y	10	Low
Beaumont 2022	Y	Y	Y	Y	N	Y	CD	Y	Y	N	Y	NA	Y	N	9	Low
Benderra 2021	Y	Y	Y	Y	N	N	CD	Y	Y	N	Y	NA	Y	N	8	Low
Beurnier 2020	Y	Y	Y	Y	N	Y	CD	N	Y	N	Y	NA	Y	N	8	Low
Blayney 2022	Y	Y	Y	Y	N	Y	CD	Y	Y	N	Y	CD	Y	Y	10	Low
Brandao-Neto 2021	Y	Y	Y	Y	N	Y	CD	Y	Y	N	Y	NA	Y	N	9	Low
Bucholc 2022	Y	N	Y	Y	N	Y	CD	Y	Y	N	Y	CD	NA	Y	8	Low
Buckner 2020	Y	Y	Y	Y	N	N	CD	Y	Y	N	Y	NA	Y	N	8	Low
Bustoz-Vasquez 2021	Y	Y	Y	Y	N	N	NA	Y	Y	N	Y	NA	Y	N	8	Low

Buttenschon 2022	Y	Y	Y	Y	N	Y	CD	N	Y	N	N	CD	Y	N	7	Moderate
Camacho Moll 2023	Y	Y	N	Y	N	Y	CD	N	Y	N	Y	CD	Y	Y	8	Low
Campbell 2022	Y	Y	Y	Y	N	N	NA	Y	Y	N	Y	NA	Y	Y	9	Low
Cardinal-Fernandez 2021	Y	Y	Y	Y	N	N	CD	Y	Y	N	Y	NA	Y	N	8	Low
Cardoso 2022	Y	Y	Y	Y	N	Y	CD	Y	Y	N	Y	CD	Y	Y	10	Low
Catalano 2023	Y	Y	N	Y	N	Y	CD	Y	Y	N	Y	NA	Y	Y	9	Low
Colnago 2022	Y	Y	Y	Y	N	Y	CD	Y	Y	N	Y	NA	Y	Y	10	Low
Covino 2021	Y	Y	Y	Y	N	Y	NA	N	Y	N	Y	NA	Y	NR	8	Low
d’Arminio-Monforte 2020	Y	Y	Y	Y	N	Y	CD	Y	Y	N	Y	NA	Y	NR	9	Low
d’Etienne 2022	Y	Y	Y	Y	N	N	NA	Y	Y	N	Y	NA	Y	N	8	Low
Dandachi 2021	Y	Y	CD	N	N	N	NA	Y	Y	N	Y	NA	Y	NR	6	Moderate
Dantas 2022	Y	Y	Y	Y	N	Y	CD	Y	Y	N	Y	NA	Y	NR	9	Low
de Oliveira Lima 2022	Y	Y	Y	Y	N	Y	CD	Y	Y	N	Y	CD	Y	Y	10	Low
Di Fusco 2022	Y	Y	Y	Y	N	N	NA	Y	Y	N	Y	CD	Y	NR	8	Low
Diaz Valez 2021	Y	Y	Y	Y	N	N	NA	Y	Y	N	Y	NA	Y	NR	8	Low
Dragano 2022	Y	Y	Y	Y	N	Y	CD	Y	Y	N	Y	NA	Y	Y	10	Low

Elavarasi 2022	Y	Y	Y	Y	N	Y	CD	Y	Y	N	Y	NA	Y	Y	10	Low
Fagard 2022	Y	Y	Y	Y	N	Y	CD	Y	Y	N	Y	NA	Y	Y	10	Low
Farrar 2022	Y	Y	Y	Y	N	Y	CD	N	N	N	Y	CD	Y	N	7	Moderate
Funk 2022	Y	Y	CD	N	N	Y	CD	Y	Y	N	Y	CD	Y	Y	8	Low
Galang 2021	Y	Y	N	Y	Y	Y	N	N	Y	N	Y	Y	CD	Y	9	Low
Gani 2022	Y	Y	Y	Y	N	Y	CD	Y	Y	N	Y	N	Y	Y	10	Low
Guan 2020	Y	Y	N	N	Y	Y	N	N	Y	N	N	Y	Y	Y	8	Low
Hardelid 2022	Y	Y	Y	Y	N	Y	CD	Y	Y	N	Y	NA	Y	Y	10	Low
Hendler 2021	Y	Y	Y	CD	Y	Y	CD	Y	Y	CD	Y	Y	Y	Y	11	Low
Henkens 2022	Y	Y	Y	Y	N	Y	CD	Y	Y	N	Y	NA	Y	Y	10	Low
Hesni 2022	Y	Y	NA	Y	NA	Y	N	NA	NA	NA	Y	NA	Y	Y	8	Low
Ho 2020	N	Y	Y	Y	Y	Y	CD	Y	Y	N	Y	N/A	Y	Y	10	Low
Houvessou 2022	Y	Y	N	N	N	Y	CD	Y	N	N	Y	N	Y	Y	7	Moderate
Impouma 2022	Y	Y	NA	Y	NA	Y	N	NA	NA	NA	Y	NA	Y	Y	7	Moderate
Islam 2021	Y	Y	NA	Y	N	Y	N	Y	NA	NA	Y	NA	Y	NR	7	Moderate
Ismail 2021	Y	Y	Y	Y	NA	N	N	NA	Y	NA	Y	NA	Y	Y	8	Low
Izzy 2020	N	Y	NA	Y	NA	Y	N	NA	Y	NA	Y	NA	Y	Y	7	Moderate
Jachymek 2022	Y	Y	CD	Y	N	Y	CD	NA	Y	N	Y	CD	Y	NR	7	Moderate
Jesmani 2023	Y	Y	Y	Y	N	Y	CD	Y	Y	N	Y	CD	Y	Y	10	Low

Kammar-Garcia 2020	Y	Y	NA	Y	NA	Y	N	CD	Y	NA	Y	NA	Y	Y	8	Low
Khalifa 2022	Y	Y	N	Y	N	Y	CD	Y	Y	N	Y	NA	Y	N	8	Low
Kofahi 2022	Y	Y	Y	Y	N	CD	N	Y	Y	N	Y	CD	Y	Y	9	Low
Kokoszka-Bargiel 2022	Y	Y	Y	Y	N	Y	N	Y	Y	N	Y	NA	Y	Y	10	Low
Koyyada 2022	Y	Y	NR	Y	NA	Y	N	Y	N	NA	Y	NA	Y	Y	8	Low
Kruger 2022	Y	Y	Y	Y	N	Y	N	Y	Y	N	Y	NA	Y	Y	10	Low
Kuhn 2022	Y	Y	NA	CD	NA	Y	N	Y	NA	NA	Y	NA	Y	Y	7	Moderate
Kumar 2021	Y	Y	NA	Y	Y	Y	N	Y	CD	NA	Y	NA	Y	N	8	Low
Lam 2022	Y	Y	Y	Y	N	Y	CD	Y	Y	N	Y	NA	Y	Y	10	Low
Larsson 2020	Y	Y	Y	Y	N	CD	NA	N	Y	N	Y	N	Y	N	7	Moderate
Levy 2022	Y	Y	Y	Y	N	Y	NA	Y	Y	N	Y	CD	Y	NR	9	Low
Lota-Salvado 2023	Y	Y	Y	Y	N	NA	NA	N	Y	N	Y	NA	Y	NR	7	Moderate
Marengoni 2021	Y	Y	Y	Y	N	Y	NA	N	Y	N	Y	CD	Y	NR	8	Low
Marin-Gomez 2022	Y	Y	Y	Y	NA	Y	NA	Y	Y	N	Y	CD	Y	NR	9	Low
Marti-Pastor 2023	Y	Y	Y	Y	NA	Y	NA	Y	Y	N	Y	CD	Y	Y	10	Low
Mertens 2022	Y	Y	Y	Y	N	Y	NA	Y	Y	N	Y	NR	Y	CD	9	Low

Mi 2020	N	Y	CD	Y	N	CD	NA	Y	Y	N	Y	NA	Y	NR	6	Moderate
Mohammadifard 2022	Y	Y	Y	Y	N	Y	NA	Y	Y	N	Y	CD	Y	Y	10	Low
Monari 2022	Y	Y	CD	Y	N	Y	NA	Y	Y	N	Y	CD	Y	NR	8	Low
Ngere 2022	Y	Y	CD	Y	N	Y	CD	Y	Y	N	Y	CD	Y	CD	8	Low
Oliveira 2023	Y	Y	Y	Y	N	Y	NA	Y	Y	N	Y	NR	Y	NR	9	Low
Pinzon 2023	Y	N	CD	Y	N	Y	NA	Y	Y	N	Y	CD	Y	Y	8	Low
Polverino 2020	Y	Y	Y	Y	N	N	NA	Y	Y	N	Y	NA	Y	Y	9	Low
Quenzer 2022	Y	Y	Y	Y	N	Y	NA	Y	Y	N	Y	CD	Y	N	9	Low
Rainer 2022	Y	Y	Y	Y	N	Y	NA	N	Y	N	Y	CD	Y	Y	9	Low
Rana 2023	Y	Y	Y	Y	N	Y	CD	N	Y	N	Y	NR	Y	NR	8	Low
Rando 2023	Y	Y	CD	Y	N	Y	NA	N	Y	N	Y	CD	Y	N	7	Moderate
Rossi 2020	Y	Y	Y	Y	N	Y	NA	Y	Y	N	Y	NA	Y	Y	10	Low
Semenzato 2021	Y	Y	Y	Y	N	N	NA	Y	Y	N	Y	NA	Y	Y	9	Low
Siddiqi 2022	Y	Y	CD	Y	N	CD	NA	Y	Y	N	Y	NR	Y	NR	7	Moderate
Simoes 2022	Y	Y	CD	N	N	Y	NA	N	Y	N	Y	NR	Y	N	6	Moderate
Siqueira 2022	Y	Y	Y	Y	N	Y	NA	Y	Y	N	Y	CD	Y	Y	10	Low
Skarbinski 2022	Y	Y	Y	Y	N	Y	NA	CD	Y	N	Y	CD	Y	Y	9	Low
Smith 2022	Y	Y	Y	Y	N	Y	CD	Y	Y	N	Y	CD	Y	N	9	Low

Solanki 2022	Y	Y	Y	Y	N	Y	NA	Y	Y	N	Y	CD	Y	Y	10	Low
Subramaniam 2022	Y	Y	CD	Y	N	Y	CD	Y	Y	N	Y	NR	Y	Y	9	Low
Sundaram 2022	Y	Y	CD	Y	N	Y	NA	Y	Y	N	Y	NR	Y	Y	9	Low
Surendra 2023	Y	Y	CD	Y	N	Y	NA	Y	CD	N	Y	CD	Y	Y	8	Low
Traiber 2022	Y	Y	N	Y	N	Y	NA	NR	CD	N	Y	CD	Y	N	6	Moderate
Toofan 2021	Y	Y	Y	Y	N	N	NA	Y	N	N	Y	NA	Y	NR	7	Moderate
Traiber 2022	Y	Y	CD	Y	N	CD	NA	NA	Y	N	Y	Y	NA	N	6	Moderate
Wiley 2022	Y	Y	CD	Y	N	Y	CD	NR	Y	N	Y	NR	Y	Y	8	Low
Zhang C 2022	Y	N	CD	Y	N	Y	NA	Y	CD	N	Y	NR	Y	NR	6	Moderate
Zhang Y 2022	Y	N	CD	Y	N	Y	NA	Y	Y	N	Y	NR	Y	Y	8	Low

S5: SUMMARY OF RESPONSES FOR RISK OF BIAS ASSESSSMENT WITH NIH TOOL

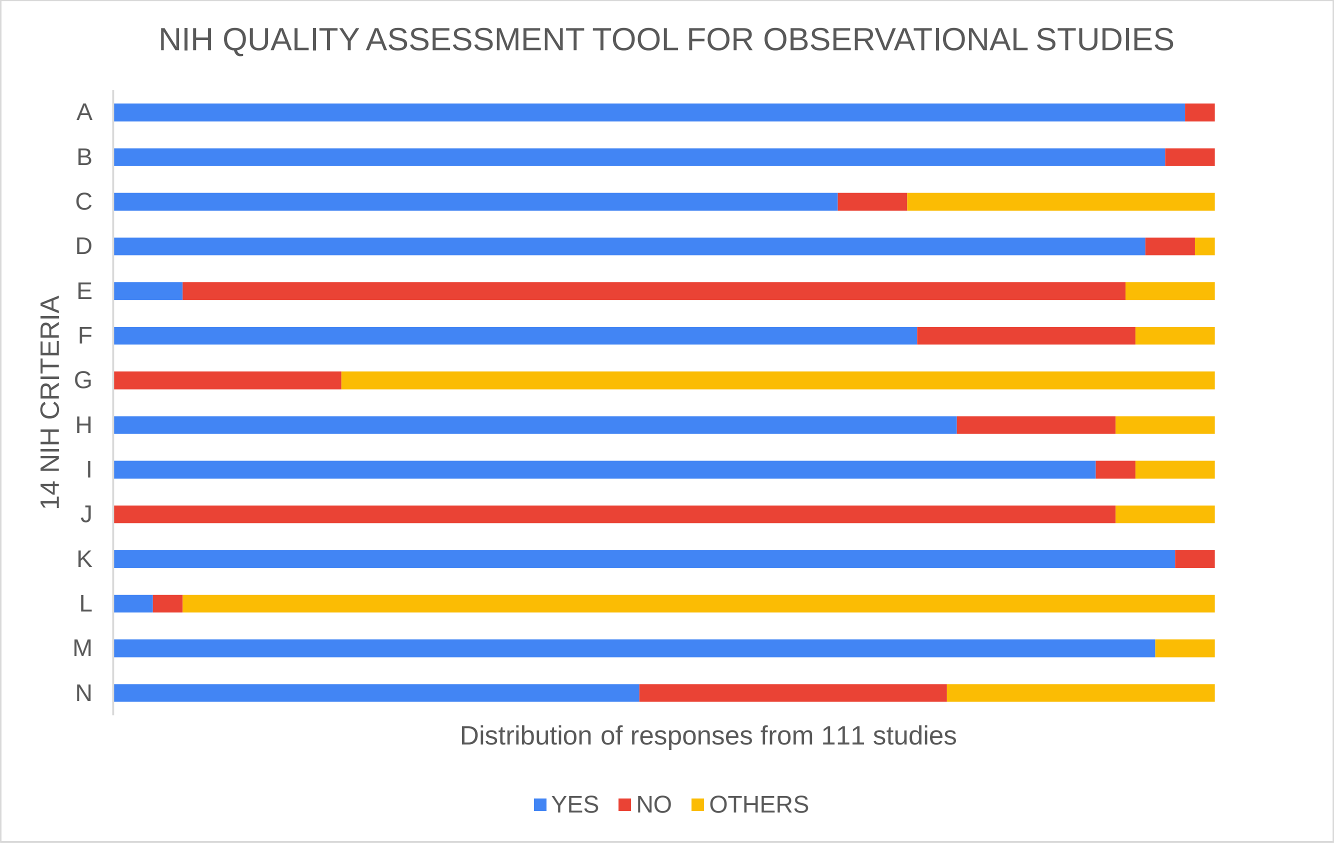

S6: FUNNEL PLOTS FOR ASSESSMENT OF PUBLICATION BIAS

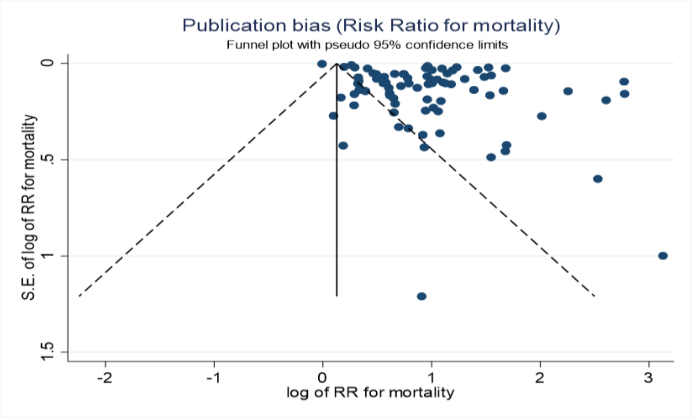

Egger’s test p= 0.00

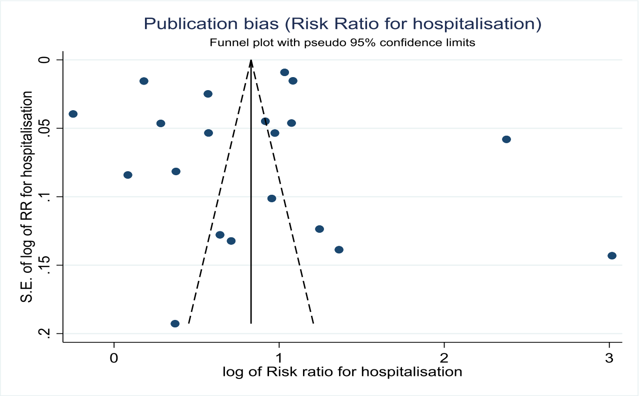

Egger’s test p= 0.76

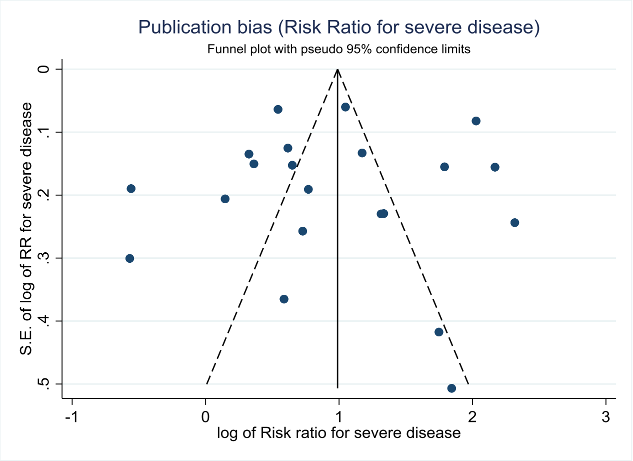

Egger's test p= 0.85

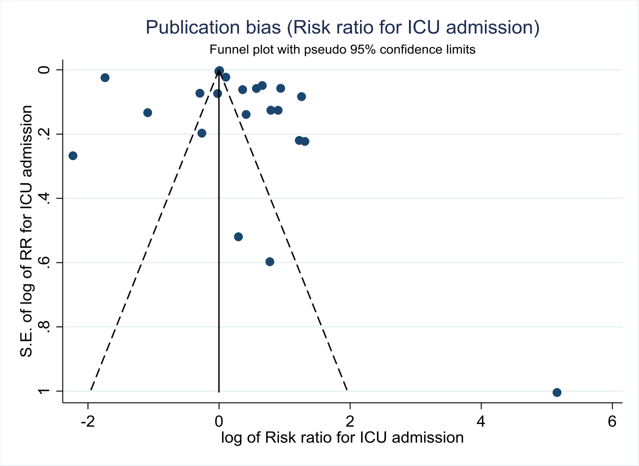

Egger's test p= 0.89

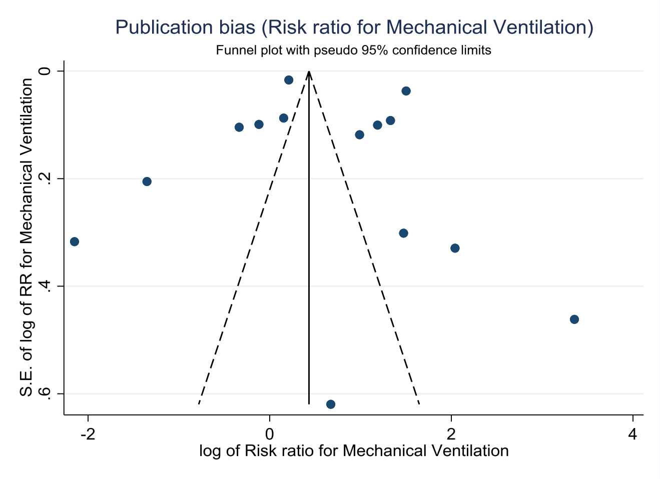

Egger's test p= 0.56

S7: FOREST PLOTS FOR COVID-19-RELATED OUTCOMES

a. Forest plot or risk ratio for mortality in children and young people vs all ages

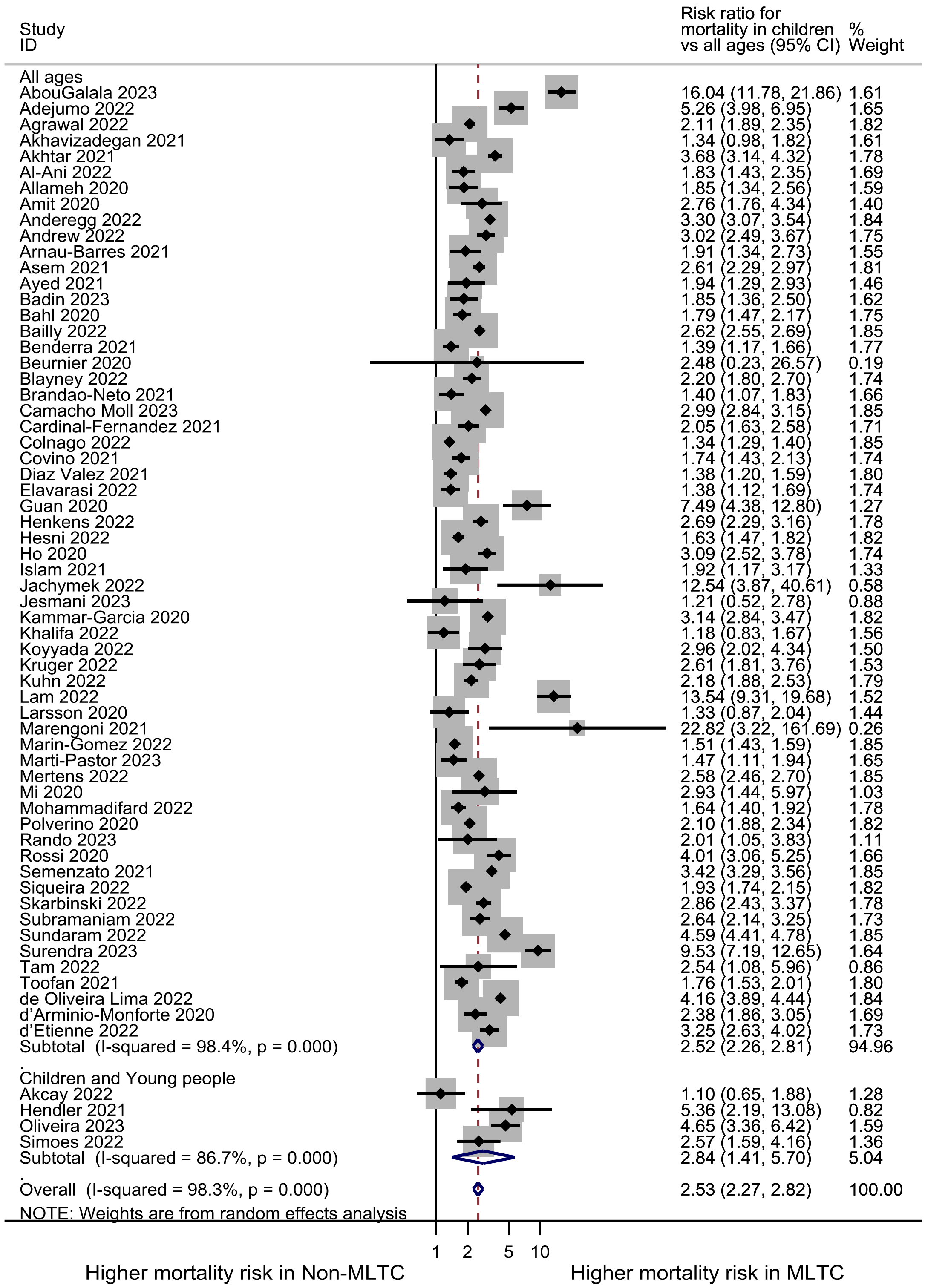

b. Forest plot of risk ratio for hospitalisation

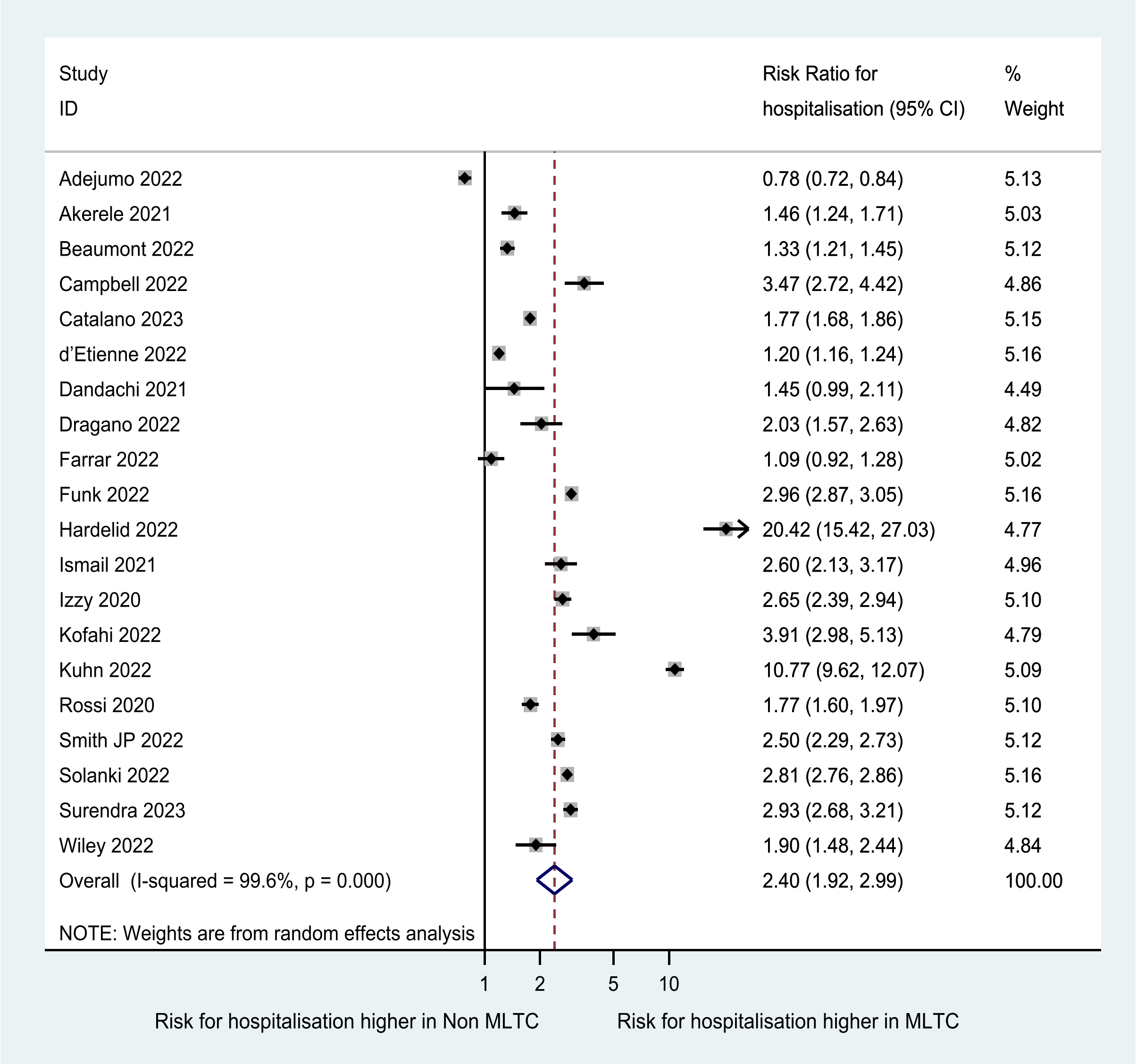

c. Forest plot of risk of hospitalisation in studies with children and young people vs all ages

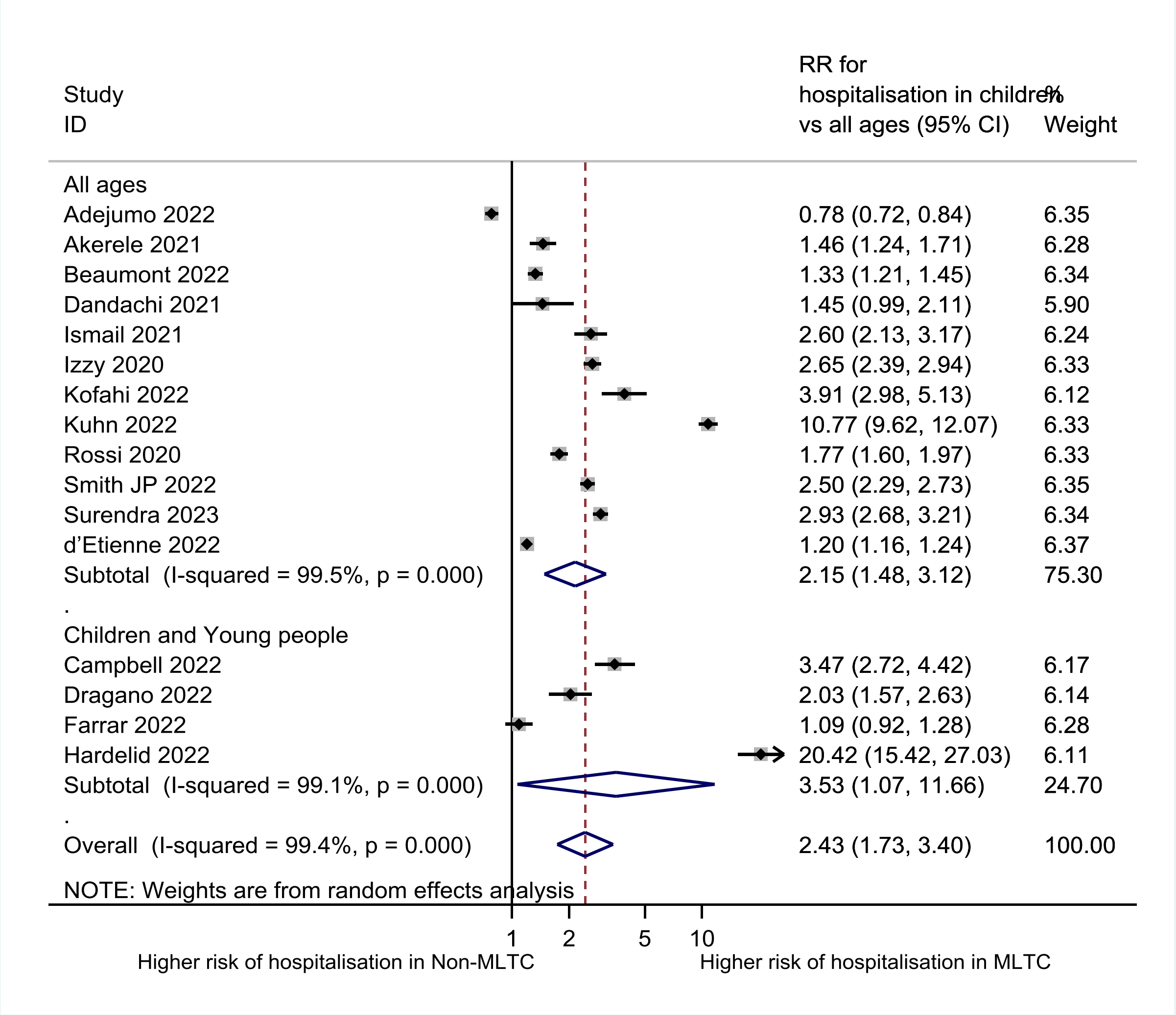

d. Forest plot for risk ratio for severe disease

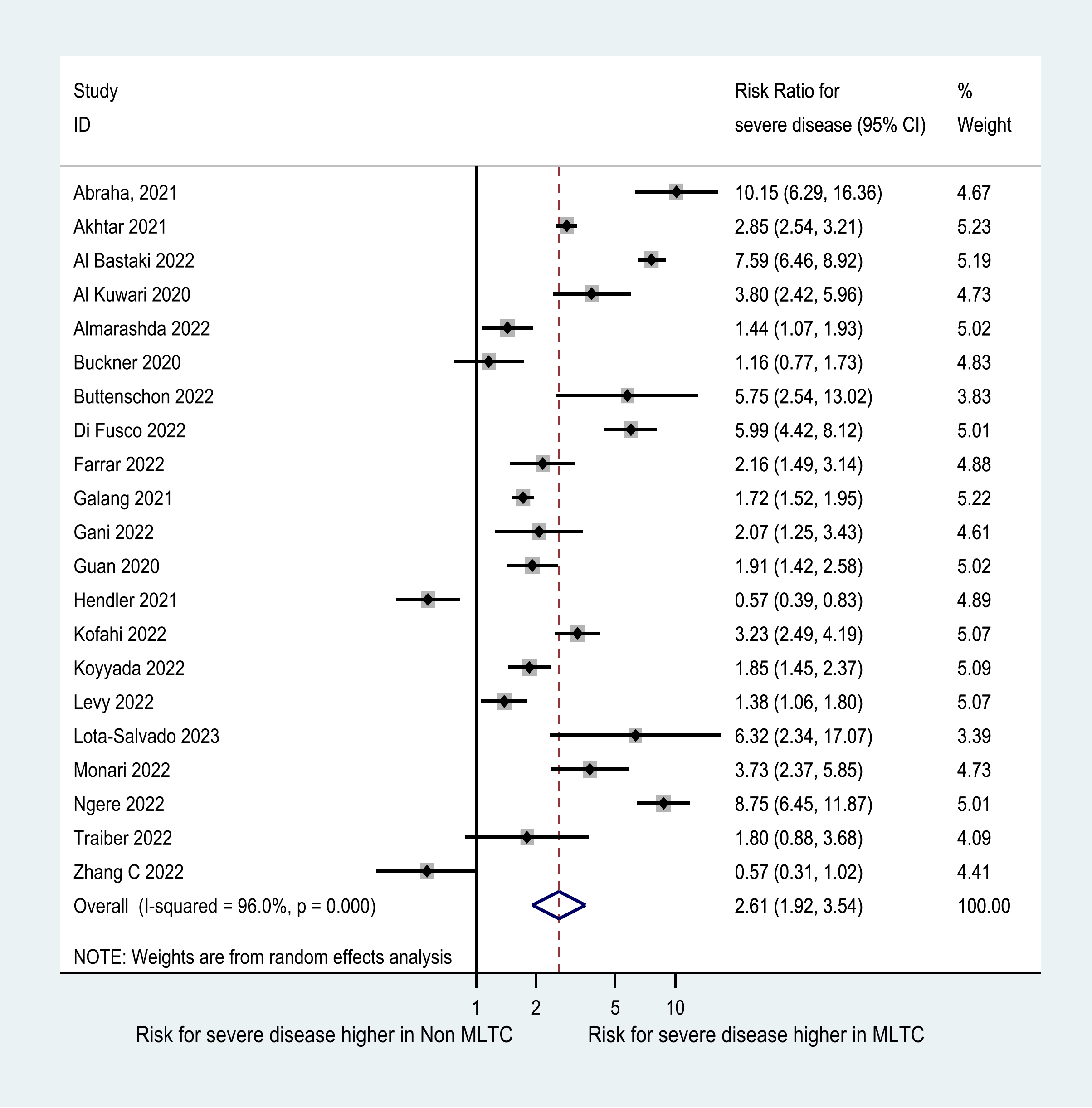

e. Forest plot for risk ratio for ICU admission

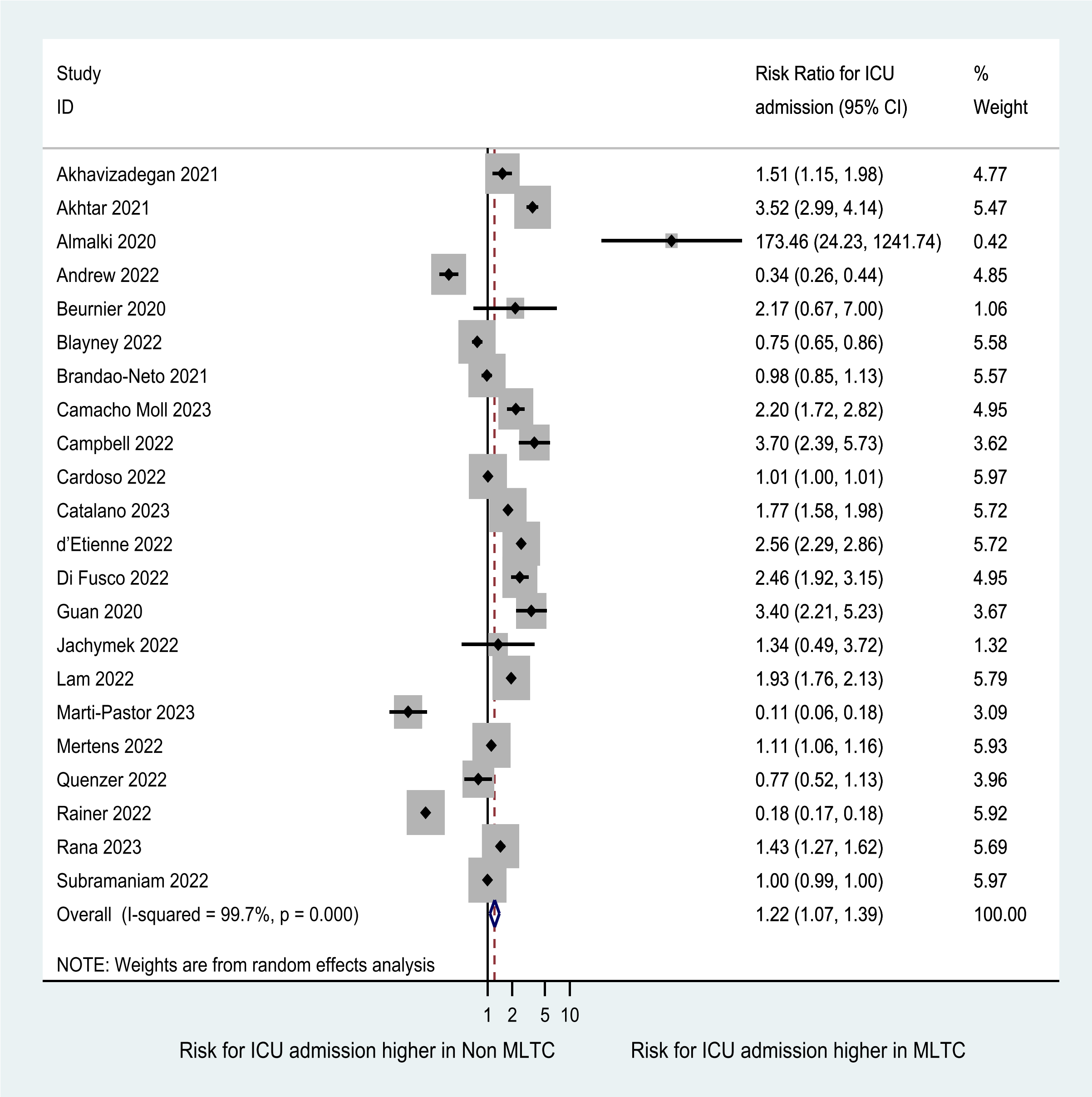

f. Forest plot for risk ratio for ICU admission in children and young people vs all ages

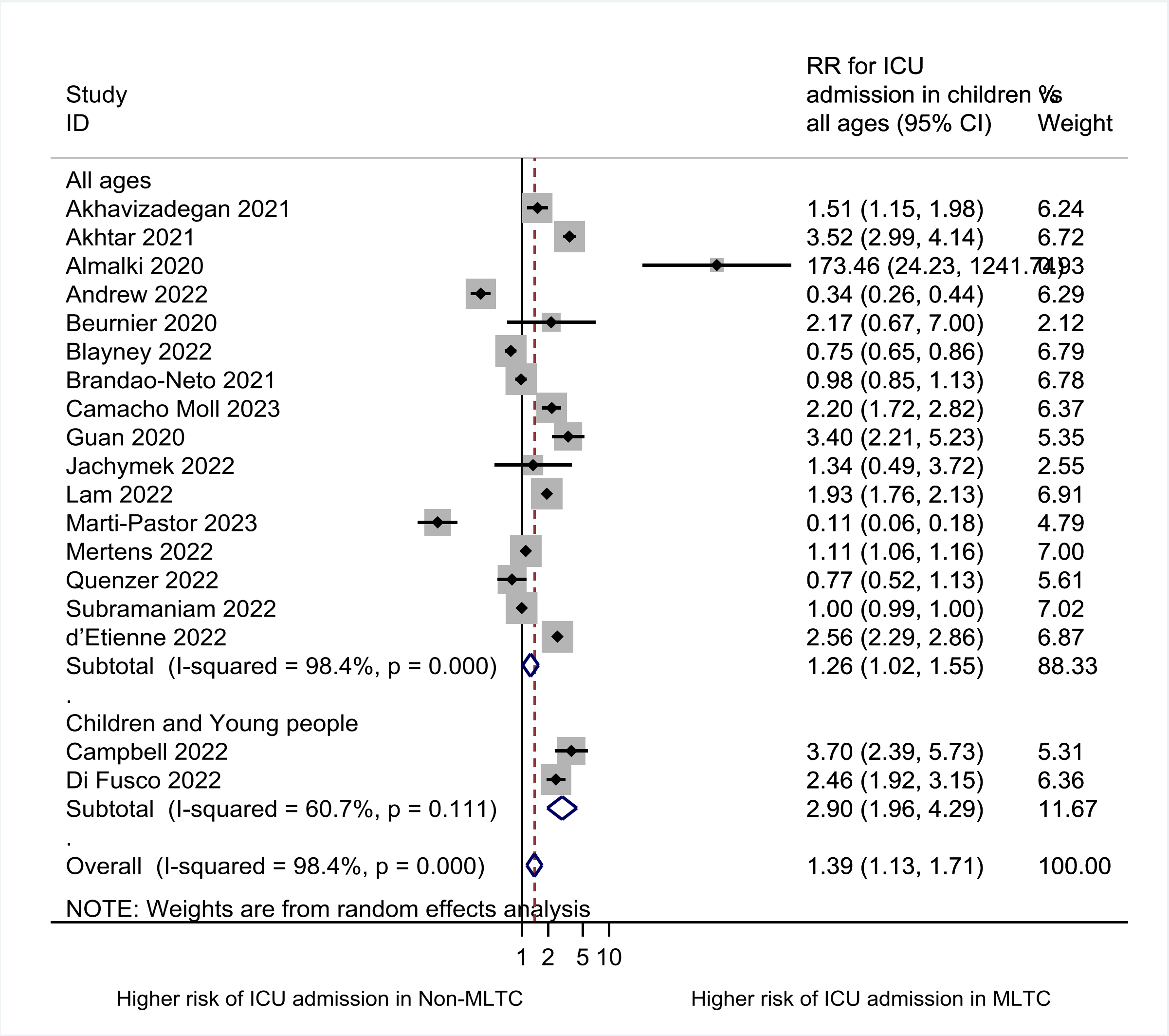

g. Forest plot for risk ratio for mechanical ventilation

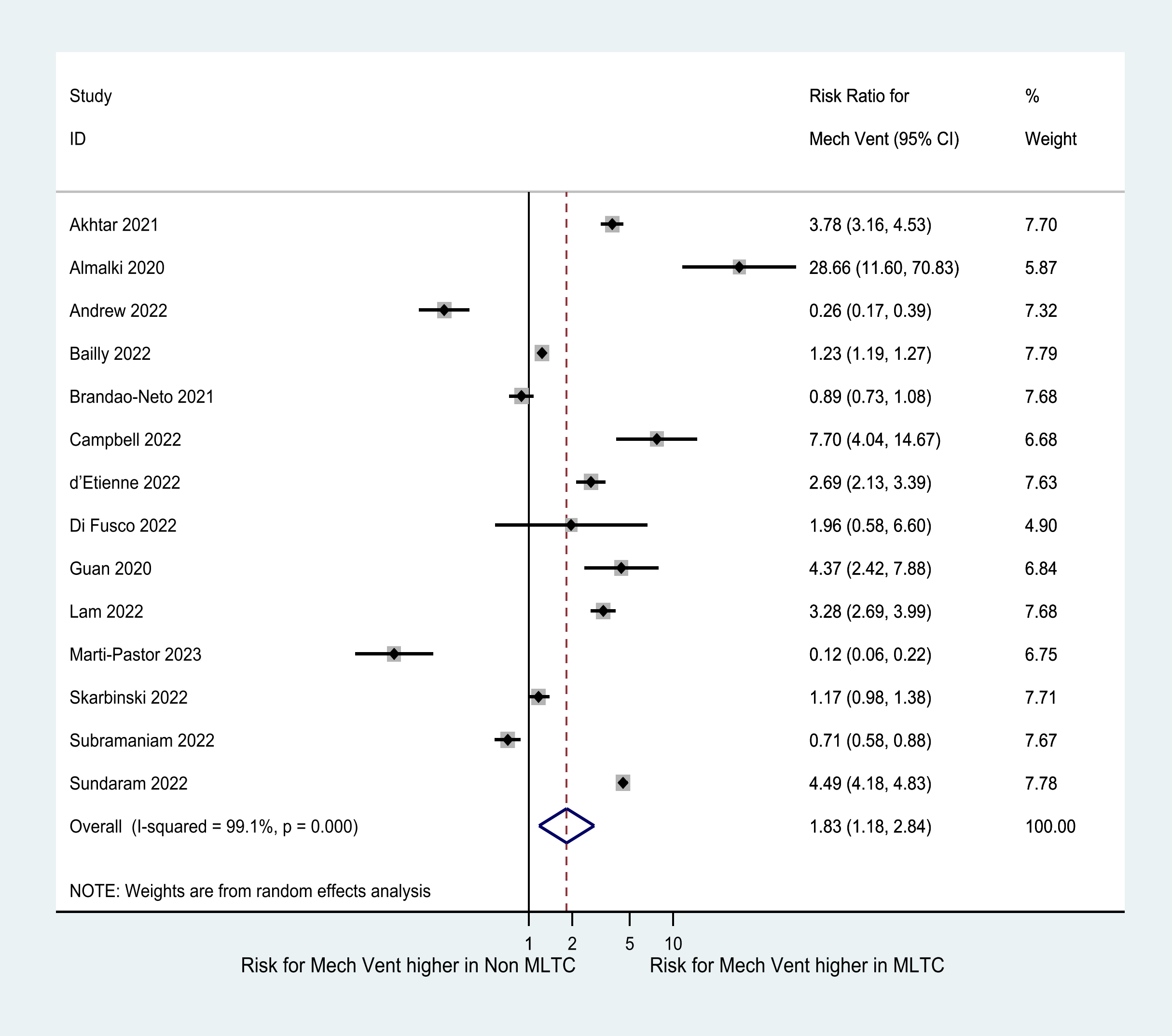

h. Forest plot for risk ratio for mechanical ventilation in children and young people vs all ages

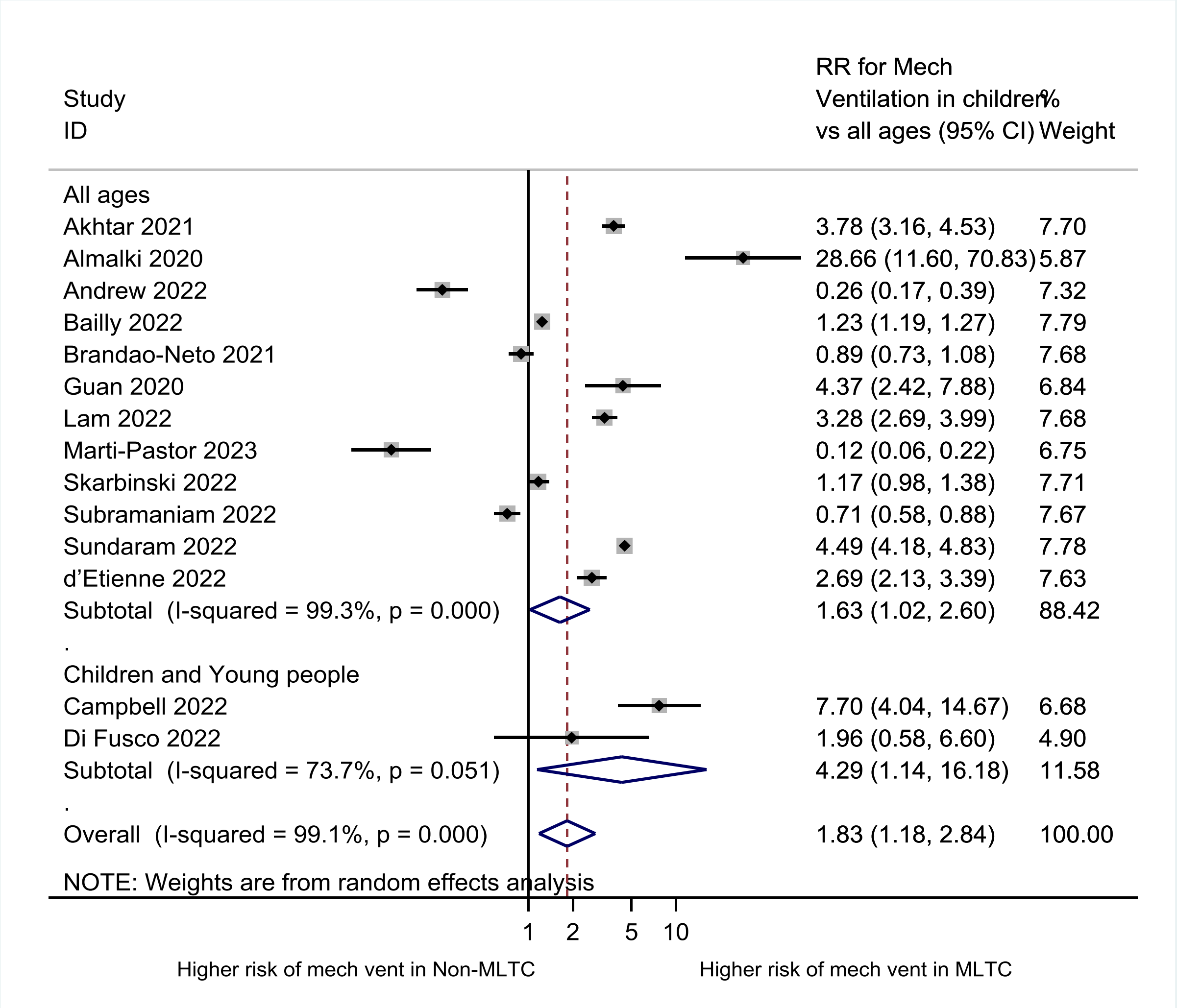

S8: FOREST PLOTS FOR SENSITIVITY ANALYSES (STUDY QUALITY)

a. Forest plot showing risk ratio for mortality by study quality (risk of bias)

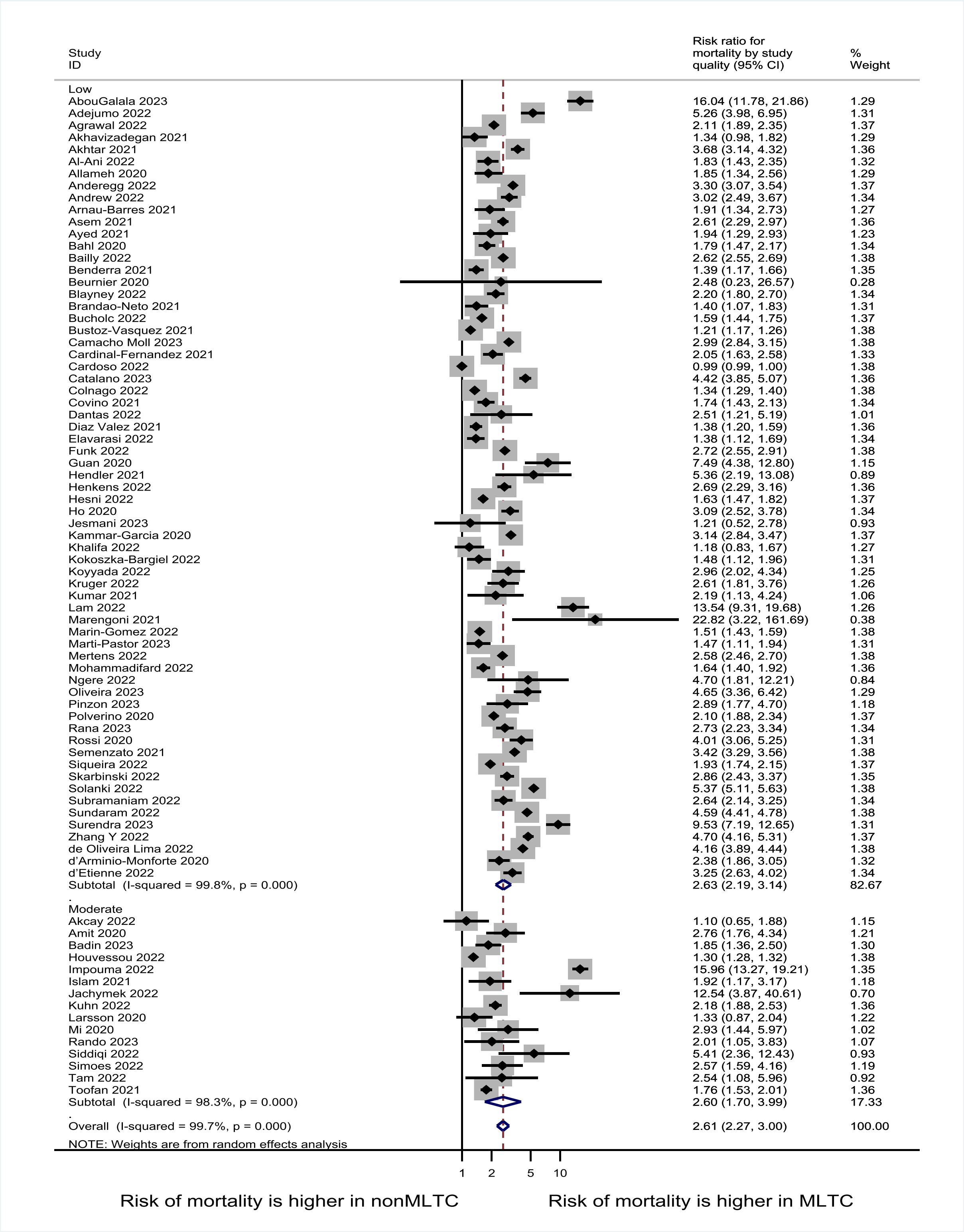

b. Forest plot showing risk ratio for hospitalisation by study quality

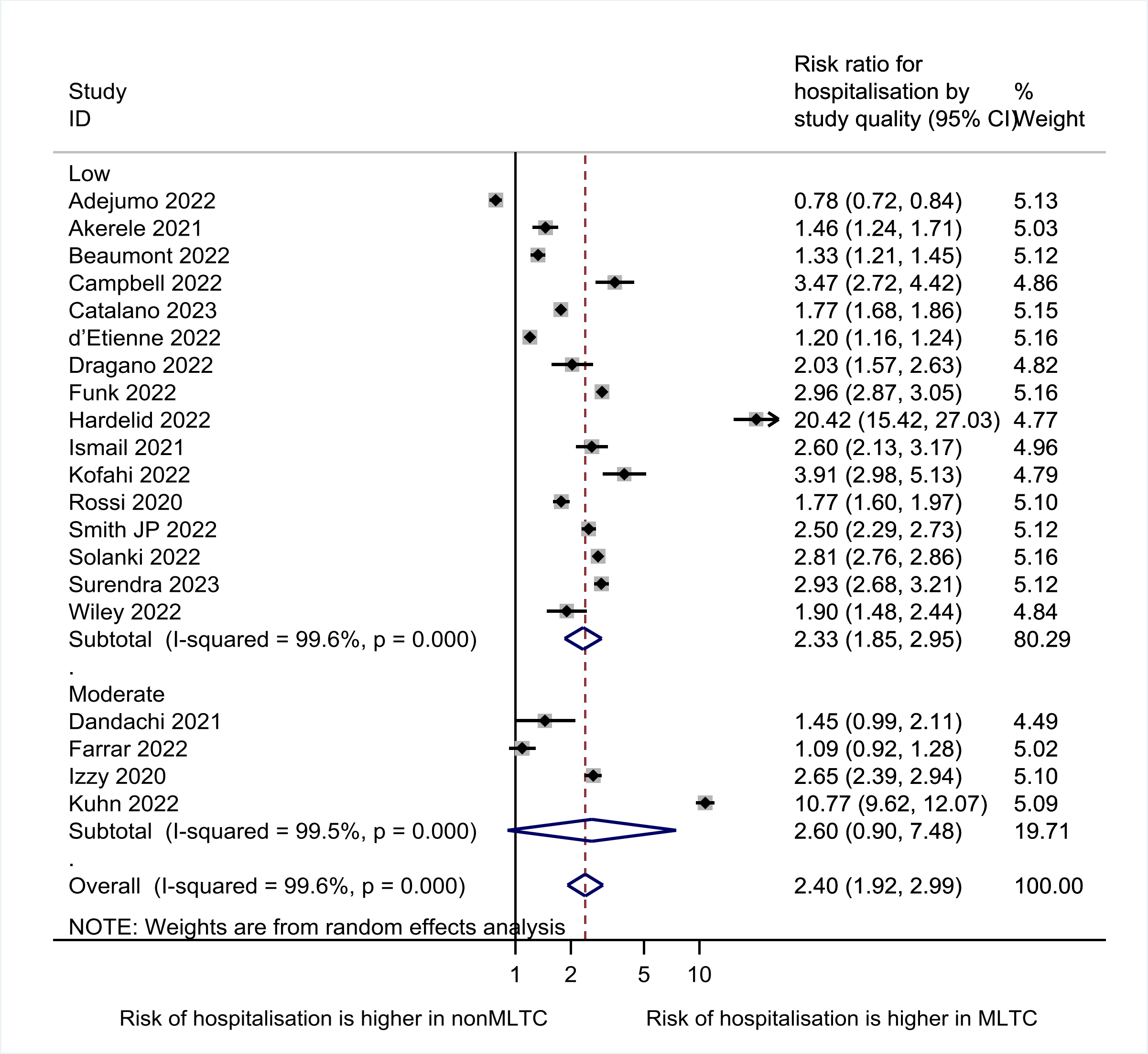

c. Forest plot showing risk ratio for severe disease by study quality

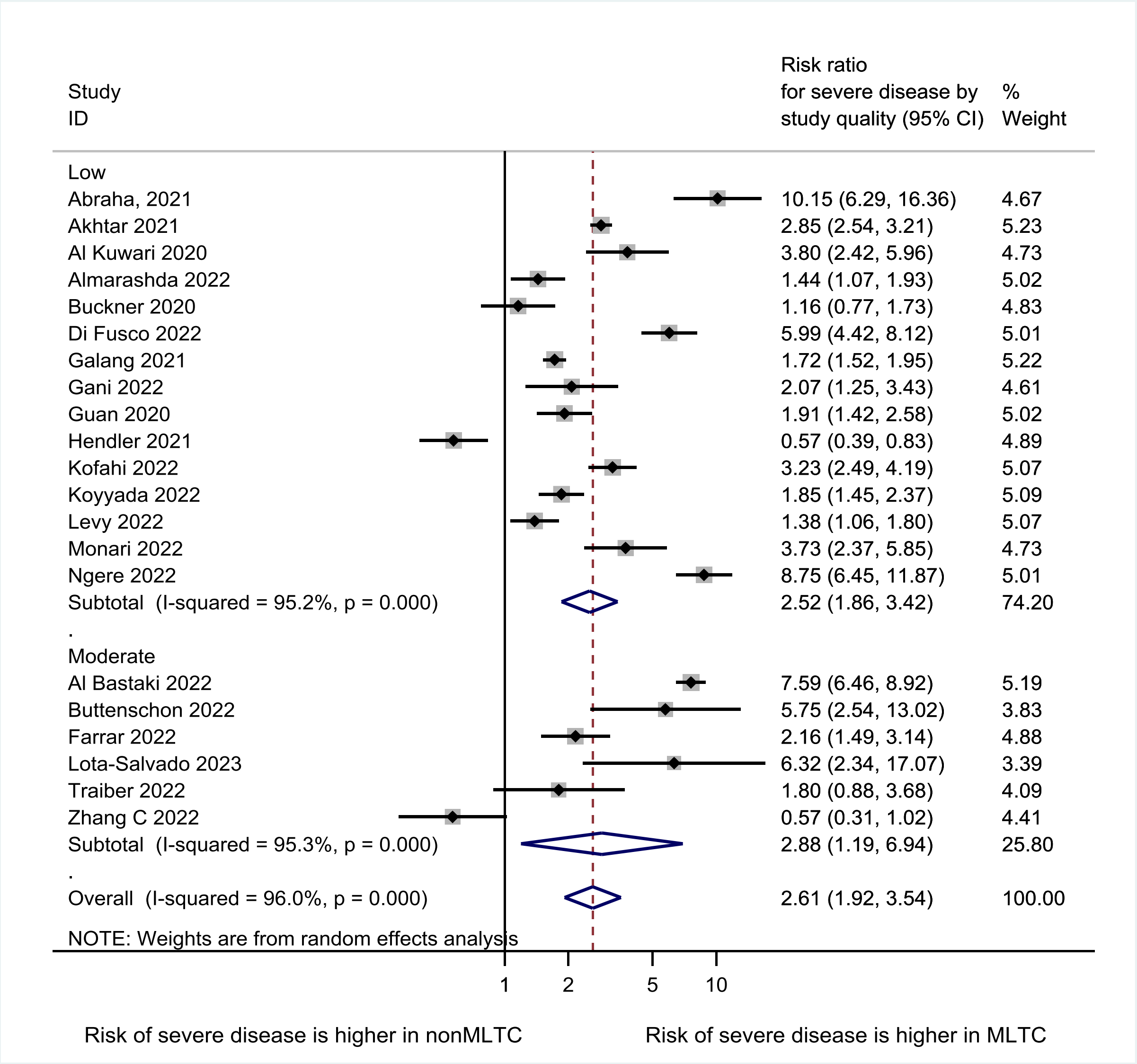

d. Forest plot showing risk ratio for ICU admission by study quality

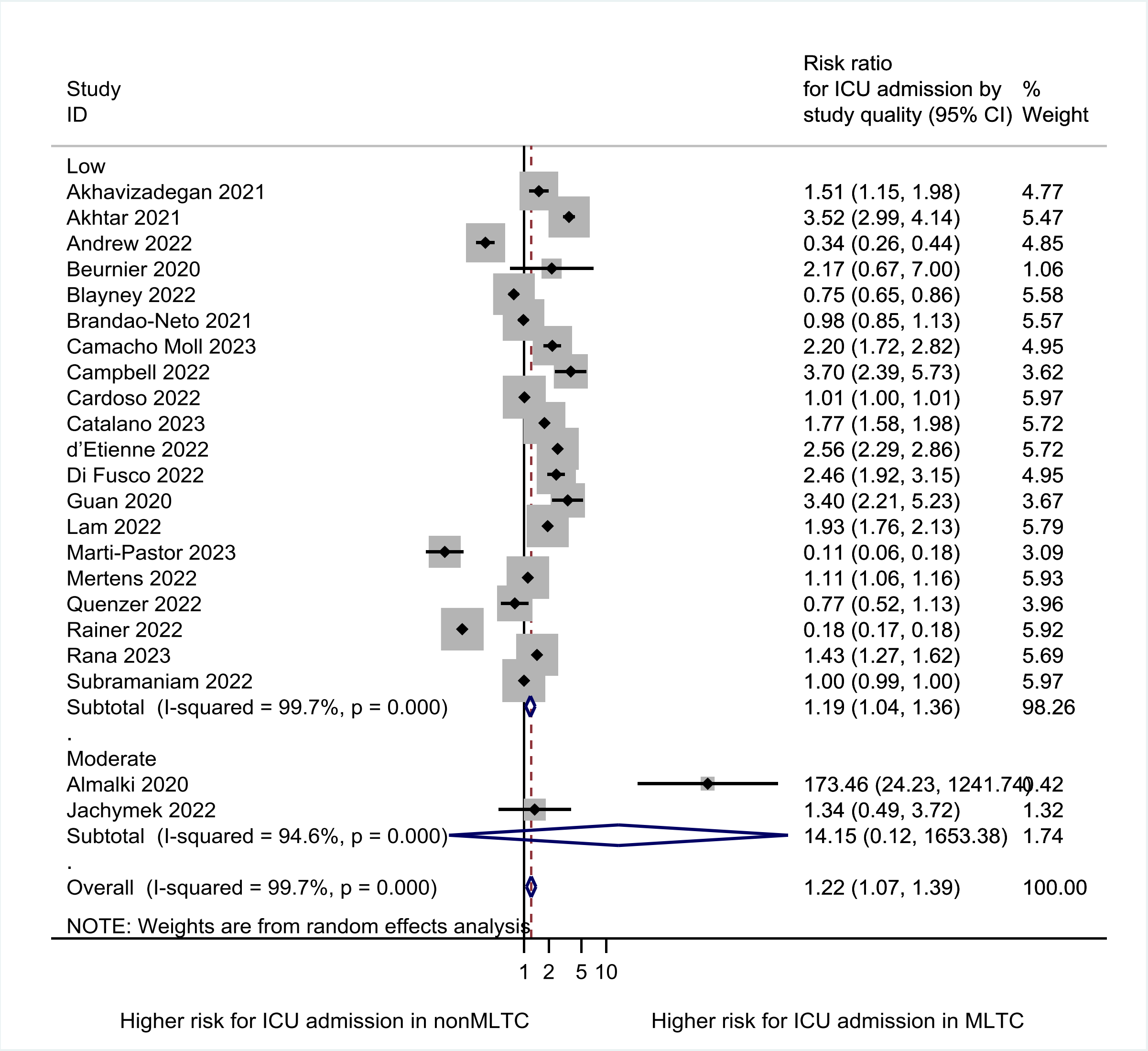

e. Forest plot showing risk ratio for Mechanical ventilation by study quality.

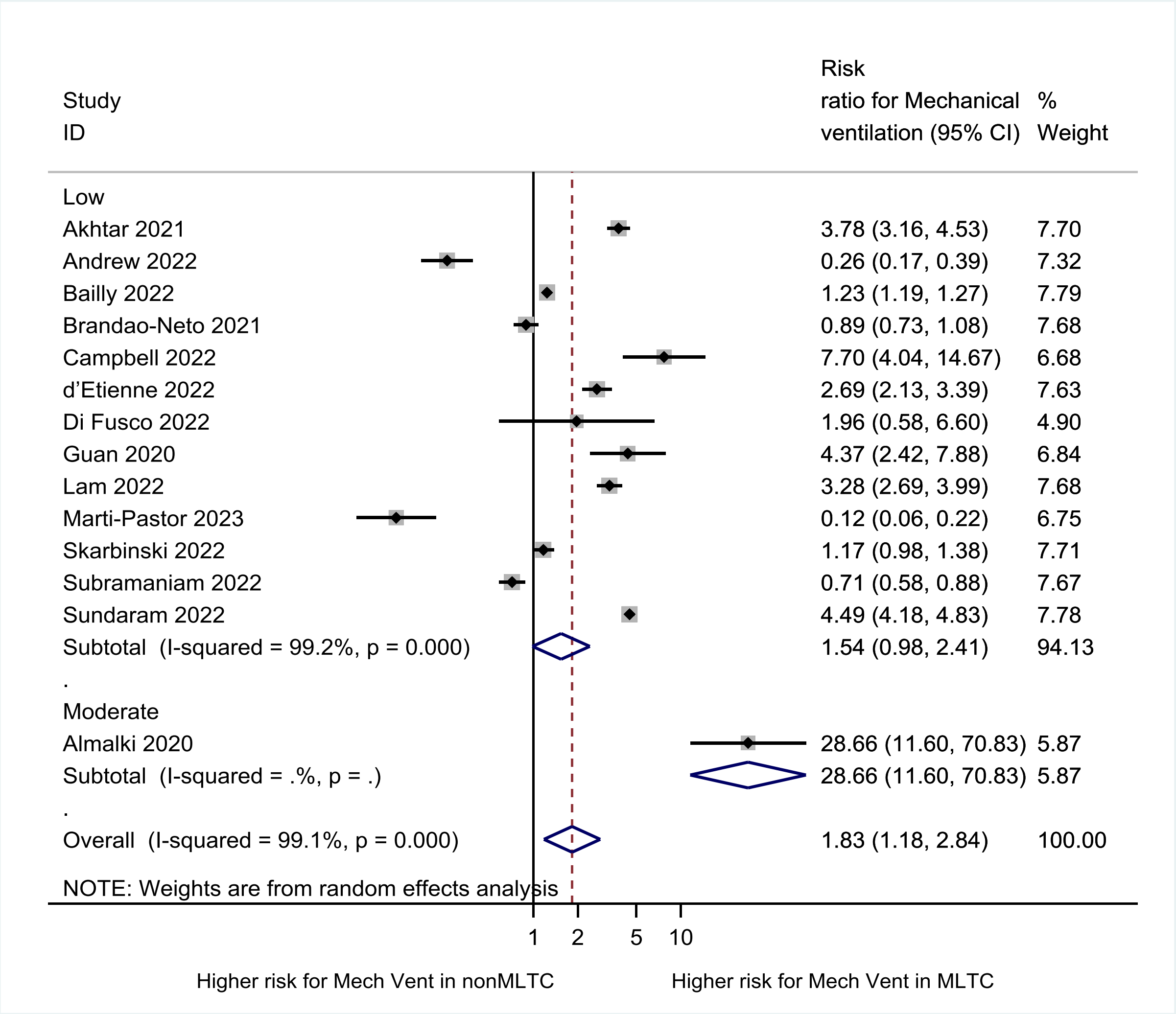

S9: Tables showing summary of sub-group analysis for pooled risk estimates for COVID-19-related outcomes.

a: Summary of subgroup analysis for RR for mortality

Variable	Pooled risk estimates and (95% Confidence intervals)	Number of studies included in analysis
**Country income group**		
Low income	15.96 (13.27 to 19.21)	1
Lower-middle income	2.5 (1.97 to 3.17)	17
Upper-middle income	2.3 (1.88 to 2.81)	24
High income	2.66 (2.35 to 3.0)	38
**MLTC measure**		
2 or more	2.47 (2.13 to 2.86)	61
3 or more	2.61 (1.28 to 5.31)	4
CFS≥ 5	2.56 (1.77 to 3.71)	7
CCI ≥ 2	3.54 (2.66 to 4.7)	8
**Study duration**		
≤ 7 months	2.70 (2.36 to 3.1)	54
> 7 months	2.41 (1.87 to 3.11)	24
**Study quality (Risk of bias)**		
Low Risk of Bias	2.63 (2.19 to 3.14)	65
Moderate Risk of Bias	2.6 (1.7 to 3.99)	15

b: Summary of subgroup analysis for RR for hospitalisation

Variable	Pooled risk estimates and (95% Confidence intervals)	Number of studies included in analysis
**Country income group**		
Lower middle income	1.89 (0.86 to 4.18)	4
Upper middle income	2.81 (2.76 to 2.86)	1
High-income	2.53 (1.89 to 3.4)	15
**MLTC measure**		
2 or more	2.44 (1.85 to 3.21)	15
3 or more	3.15 (2.16 to 4.6)	2
CCI ≥ 2	1.77 (1.7 to 1.85)	3
**Study duration**		
≤ 7 months	2.05 (1.55 to 2.69)	12
> 7 months	3.05 (1.94 to 4.78)	8
**Study quality (Risk of bias)**		
Low Risk of Bias	2.33 (1.85 to 2.95)	16
Moderate Risk of Bias	2.6 (0.9 to 7.48)	4

c: Summary of Subgroup analysis for RR severe disease

Variable	Pooled risk estimates and (95% Confidence intervals)	Number of studies included in analysis
**Country income group**		
Low income	10.15 (6.,29 to 16.36)	1
Lower middle income	3.71 (2.32 to 5.94)	5
Upper middle income	1.18 (0.63 to 2.19)	5
High income	2.78 (1.69 to 4.58)	10
**MLTC measure**		
2 or more	2.73 (1.86 to 4)	15
3 or more	2.4 (1.18 to 4.86)	4
CCI ≥ 2	2.23 (0.85 to 5.89)	2
**Study duration**		
≤ 7 months	2.87 (1.98 to 4.14)	11
> 7 months	2.37 (1.37 to 4.12)	10
**Study quality (Risk of bias)**		
Low Risk of Bias	2.52 (1.86 to 3.24)	15
Moderate Risk of Bias	2.88 (1.19 to 6.94)	6

d: Summary of Subgroup analysis for RR ICU admission

Variable	Pooled risk estimates and (95% Confidence intervals)	Number of studies included in analysis
**Country income group**		
Lower middle income	1.97 (1.05 to 3.69)	3
Upper middle income	1.37 (0.99 to 1.89)	5
High-income	1.17 (0.75 to 1.81)	14
**MLTC measure**		
2 or more	1.66 (1.36 to 2.01)	14
3 or more	2.46 (1.92 to 3.15)	1
CFS≥ 5	0.45 (0.17 to 1.19)	4
CCI ≥ 2	0.84 (0.14 to 5.1)	3
**Study duration**		
≤ 7 months	1.78 (1.25 to 2.55)	13
> 7 months	0.90 (1.07 to 1.39)	9
**Study quality (Risk of bias)**		
Low Risk of Bias	1.19 (1.04 to 1.36)	20
Moderate Risk of Bias	14.15 (0.12 to 1653.38)	2

e: Summary of Subgroup analysis for RR Mechanical ventilation

Variable	Pooled risk estimates and (95% Confidence intervals)	Number of studies included in analysis
**Country income group**		
Lower middle income	3.78 (3.16 to 4.53)	1
Upper middle income	1.92 (0.4 to 9.14)	2
High-income	1.69 (1.01 to 2.84)	11
**MLTC measure**		
2 or more	4.19 (2.03 to 8.61)	6
3 or more	1.96 (0.58 to 6.6)	1
CFS≥ 5	0.29 (0.1 to 0.8)	3
CCI ≥ 2	2.15 (0.96 to 4.81)	4
**Study duration**		
≤ 7 months	2.11 (1.34 to 3.30)	9
> 7 months	1.33 (0.58 to 3.06)	5
**Study quality (Risk of bias)**		
Low Risk of Bias	1.54 (0.98 to 2.41)	13
Moderate Risk of Bias	28.66 (11.6 to 70.83)	1

S10: Results of meta-regression models assessing the effects of continuous variables on the risk ratio for outcomes

OUTCOMES	REGRESSION DATA	VARIABLES	
		Average age	Proportion of males
**Mortality**	Co-efficient	-0.03	2.29
	95% CI	-0.08 to 0.02	-4.27 to 8.86
	p	0.291	0.488
**Hospitalisation**	Co-efficient	-0.10	-14.2
	95% CI	-0.24 to 0.05	-38.7 to 10.28
	p	0.166	0.239
**Severe disease**	Co-efficient	-0.03	5.37
	95% CI	-0.07 to 0.02	-1.46 to 12.21
	p	0.298	0.116
**ICU admission**	Co-efficient	-0.35	148.2
	95% CI	-1.41 to 0.71	35.46 to 260.94
	p	0.497	**0.013**
**Mechanical ventilation**	Co-efficient	-0.12	53.16
	95% CI	-0.33 to 0.10	24.66 to 81.65
	p	0.259	**0.002**

S11: Bubble plots for meta-regression for proportion of males and ICU admission, Mechanical ventilation

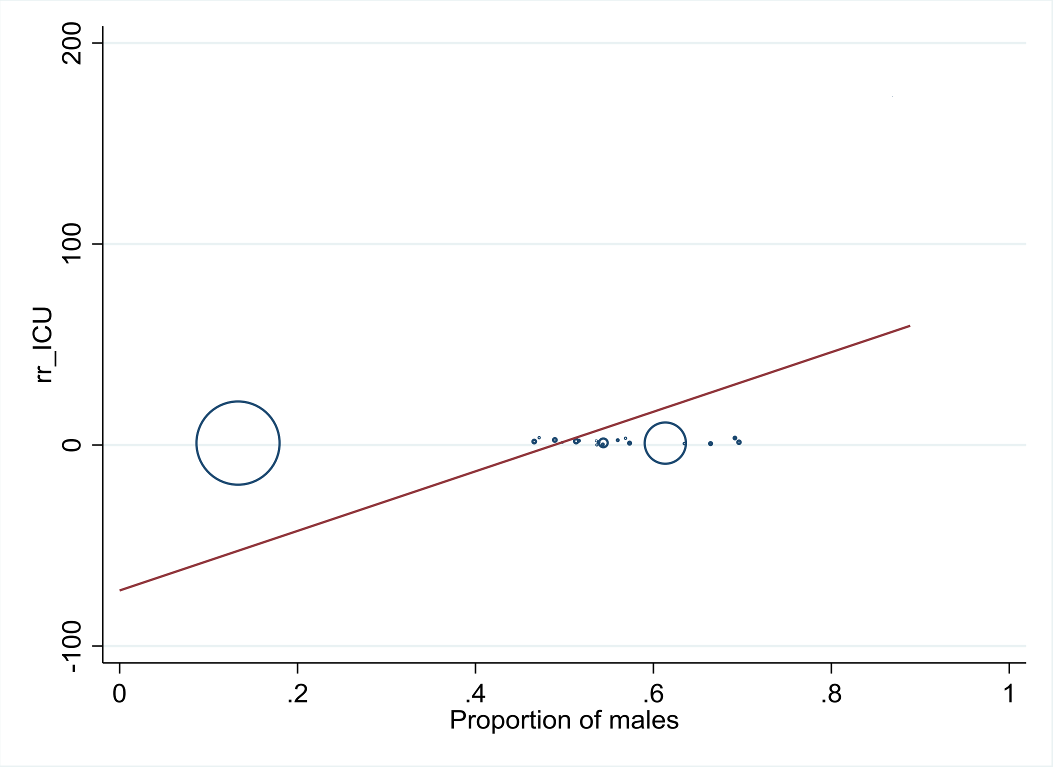

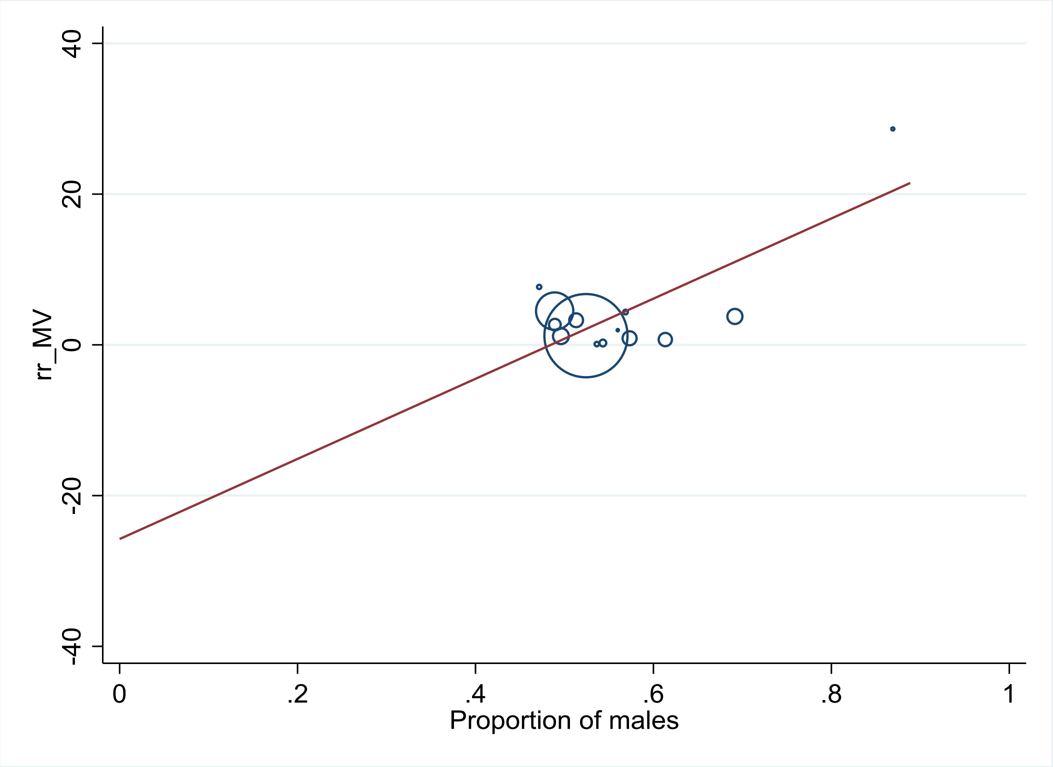

S12: LIST OF REFERENCES FOR 111 STUDIES INCLUDED IN THE SYSTEMATIC REVIEW

1. AbouGalala K, Elsayed B, Elmarasi M, Kotob M, Taha R, Abbasher F, et al. Association between Multimorbidity and COVID-19 Mortality in Qatar: A Cross-Sectional Study. Microbiol Res. 2023;14(1):289-96.

2. Abraha HE, Gessesse Z, Gebrecherkos T, Kebede Y, Weldegiargis AW, Tequare MH, et al. Clinical features and risk factors associated with morbidity and mortality among patients with COVID-19 in northern Ethiopia. International Journal of Infectious Diseases. 2021;105:776-83.

3. Accordino S, Canetta C, Bettini G, Corsico F, Ghigliazza G, Barbetta L, et al. Clinical Characteristics and Outcomes of Patients with Acute Respiratory Failure Due to SARS-CoV-2 Interstitial Pneumonia Treated with CPAP in a Medical Intermediate Care Setting: A Retrospective Observational Study on Comparison of Four Waves. Journal of Clinical Medicine. 2023;12(4).

4. Adejumo O, Ogunniyan T, Adetola A, Mba S, Ogunbayo O, Oladokun O, et al. Factors associated with death among hospitalized COVID-19 patients in Lagos State, Nigeria: a retrospective cross-sectional study. African Health Sciences. 2022;22(3):486-94.

5. Agrawal U, Azcoaga-Lorenzo A, Fagbamigbe AF, Vasileiou E, Henery P, Simpson CR, et al. Association between multimorbidity and mortality in a cohort of patients admitted to hospital with COVID-19 in Scotland. Journal of the Royal Society of Medicine. 2022;115(1).

6. Akcay N, Kihtir HS, Durak C, Kendirli T, Havan M, Kockuzu E, et al. Mortality Risk Factors Among Critically Ill Children With Acute COVID-19 in PICUs: A Multicenter Study From Turkish Pediatric Critical COVID-19 and MIS-C Study Group. Pediatr Infect Dis J. 2022;41(9):742-50.

7. Akerele IO, Oreh AC, Kawu MB, Ahmadu A, Okechukwu JN, Mbo DN, et al. Clinical presentation and hospitalisation duration of 201 coronavirus disease 2019 patients in Abuja, Nigeria. Afr J Prim Health Care Fam Med. 2021;13(1):1-8.

8. Akhavizadegan H, Aghaziarati M, Balalemi MGR, Broujeni ZA, Taghizadeh F, Arab IA, et al. Relationship Between Comorbidity, Chronic Diseases, ICU Hospitalization, and Death Rate in the Elderly With Coronavirus Infection. Iranian J Ageing. 2021;16(1):86-101.

9. Akhtar H, Khalid S, ur Rahman F, Umar M, Ali S, Afridi M, et al. Presenting Characteristics, Comorbidities, and Outcomes among Patients with COVID-19 Hospitalized in Pakistan: Retrospective Observational Study. JMIR Public Health and Surveillance. 2021;7(12).

10. Al-Ani A, Ghazzay HI, Al Shawi AF, Al-Koubaisy HNE, Al-Ani F, Aldouri M. Association of chronic diseases with mortality among hospitalized patients with COVID-19 treated with convalescent plasma: Evidence from a single center - Iraq. J Emerg Med Trauma Acute Care. 2022;2022(2).

11. Al Bastaki NA, Sheek-Hussein M, Shukla A, Al-Bluwi N, Saddik B. An Evaluation of Non-Communicable Diseases and Risk Factors Associated with COVID-19 Disease Severity in Dubai, United Arab Emirates: An Observational Retrospective Study. International journal of environmental research and public health. 2022;19(21).

12. Al Kuwari HM, Abdul Rahim HF, Abu-Raddad LJ, Abou-Samra A-B, Al Kanaani Z, Al Khal A, et al. Epidemiological investigation of the first 5685 cases of SARS-CoV-2 infection in Qatar, 28 February-18 April 2020. BMJ open. 2020;10(10):e040428.

13. Allameh SF, Nemati S, Ghalehtaki R, Mohammadnejad E, Aghili SM, Khajavirad N, et al. Clinical Characteristics and Outcomes of 905 COVID-19 Patients Admitted to Imam Khomeini Hospital Complex in the Capital City of Tehran, Iran. Archives of Iranian medicine. 2020;23(11):766-75.

14. Almalki ZS, Khan MF, Almazrou S, Alanazi AS, Iqbal MS, Alqahtani A, et al. Clinical Characteristics and Outcomes Among COVID-19 Hospitalized Patients with Chronic Conditions: A Retrospective Single-Center Study. J Multidiscip Healthc. 2020;13:1089-97.

15. Almarashda AM, Rabbani SA, Kurian MT, Cherian A. Clinical Characteristics, Risk Factors for Severity and Pharmacotherapy in Hospitalized COVID-19 Patients in the United Arab Emirates. Journal of Clinical Medicine [Internet]. 2022; 11(9).

16. Amit M, Sorkin A, Chen J, Cohen B, Karol D, Tsur AM, et al. Clinical course and outcomes of severe covid-19: A national scale study. Journal of Clinical Medicine. 2020;9(7):1-12.

17. Anderegg N, Panczak R, Egger M, Low N, Riou J. Survival among people hospitalized with COVID-19 in Switzerland: a nationwide population-based analysis. BMC Medicine. 2022;20(1).

18. Andrew MK, Godin J, LeBlanc J, Boivin G, Valiquette L, McGeer A, et al. Older Age and Frailty are Associated with Higher Mortality but Lower ICU Admission with COVID-19. Canadian Geriatrics J. 2022;25(2):183-96.

19. Arnau-Barrés I, Pascual-Dapena A, López-Montesinos I, Gómez-Zorrilla S, Sorlí L, Herrero M, et al. Severe hypoalbuminemia at admission is strongly associated with worse prognosis in older adults with sars-cov-2 infection. Journal of Clinical Medicine. 2021;10(21).

20. Asem N, Hassany M, Taema K, Masoud H, Elassal G, Kamal E, et al. Demographic and clinical features associated with in-hospital mortality in egyptian covid-19 patients: A retrospective cohort study. Open Access Macedonian Journal of Medical Sciences. 2021;9:1068-75.

21. Ayed M, Borahmah AA, Yazdani A, Sultan A, Mossad A, Rawdhan H. Assessment of Clinical Characteristics and Mortality-Associated Factors in COVID-19 Critical Cases in Kuwait. Med Princ Pract. 2021;30(2):185-92.

22. Badin RC, Amorim RLOD, Aguila A, Manaças LRA. Clinical and pharmacological factors associated with mortality in patients with COVID-19 in a high complexity hospital in Manaus: A retrospective study. PLoS ONE. 2023;18(2 February).

23. Bahl A, Van Baalen MN, Ortiz L, Chen NW, Todd C, Milad M, et al. Early predictors of in-hospital mortality in patients with COVID-19 in a large American cohort. Internal and Emergency Medicine. 2020;15(8):1485-99.

24. Bailly L, Fabre R, Courjon J, Carles M, Dellamonica J, Pradier C. Obesity, diabetes, hypertension and severe outcomes among inpatients with coronavirus disease 2019: a nationwide study. Clinical microbiology and infection : the official publication of the European Society of Clinical Microbiology and Infectious Diseases. 2022;28(1):114-23.

25. Beaumont AL, Vignes D, Sterpu R, Bussone G, Kansau I, Pignon C, et al. Factors associated with hospital admission and adverse outcome for COVID-19: Role of social factors and medical care. Infectious Diseases Now. 2022.

26. Benderra MA, Aparicio A, Leblanc J, Wassermann D, Kempf E, Galula G, et al. Clinical characteristics, care trajectories and mortality rate of sars-cov-2 infected cancer patients: A multicenter cohort study. Cancers. 2021;13(19).

27. Beurnier A, Jutant EM, Jevnikar M, Boucly A, Pichon J, Preda M, et al. Characteristics and outcomes of asthmatic patients with COVID-19 pneumonia who require hospitalisation. European Respiratory Journal. 2020;56(5).

28. Blayney MC, Stewart NI, Kaye CT, Puxty K, Chan Seem R, Donaldson L, et al. Prevalence, characteristics, and longer-term outcomes of patients with persistent critical illness attributable to COVID-19 in Scotland: a national cohort study. British Journal of Anaesthesia. 2022;128(6):980-9.

29. Brandao Neto RA, Marchini JF, Marino LO, Alencar JCG, Neto FL, Ribeiro S, et al. Mortality and other outcomes of patients with coronavirus disease pneumonia admitted to the emergency department: A prospective observational Brazilian study. PLoS ONE. 2021;16(1).

30. Bucholc M, Bradley D, Bennett D, Patterson L, Spiers R, Gibson D, et al. Identifying pre-existing conditions and multimorbidity patterns associated with in-hospital mortality in patients with COVID-19. Scientific reports. 2022;12(1):17313.

31. Buckner FS, McCulloch DJ, Atluri V, Blain M, McGuffin SA, Nalla AK, et al. Clinical Features and Outcomes of 105 Hospitalized Patients with COVID-19 in Seattle, Washington. Clinical Infectious Diseases. 2020;71(16):2167-73.

32. Bustos-Vázquez E, Padilla-González E, Reyes-Gómez D, Carmona-Ramos MC, Monroy-Vargas JA, Benítez-Herrera AE, et al. Survival of COVID-19 with Multimorbidity Patients. Healthcare 2021, Vol 9, Page 1423. 2021;9(11):1423-.

33. Buttenschon HN, Lynggaard V, bol SG, Glassou EN, Haagerup A. Comparison of the clinical presentation across two waves of COVID-19: a retrospective cohort study. BMC infectious diseases. 2022;22(1):423.

34. Camacho Moll ME, Mata Tijerina VL, Silva Ramírez B, Peñuelas Urquides K, González Escalante LA, Escobedo Guajardo BL, et al. Sex, Age, and Comorbidities Are Associated with SARS-CoV-2 Infection, COVID-19 Severity, and Fatal Outcome in a Mexican Population: A Retrospective Multi-Hospital Study. Journal of Clinical Medicine. 2023;12(7).

35. Campbell JI, Dubois MM, Savage TJ, Hood-Pishchany MI, Sharma TS, Petty CR, et al. Comorbidities Associated with Hospitalization and Progression Among Adolescents with Symptomatic Coronavirus Disease 2019. The Journal of pediatrics. 2022;245:102-10.e2.

36. Cardinal-Fernández P, Cuesta EG, Barberán J, Varona JF, Estirado A, Moreno A, et al. Clinical characteristics and outcomes of 1,331 patients with covid-19: Hm spanish cohort. Rev Esp Quimioter. 2021;34(4):342-52.

37. Cardoso JP, Calazans MIP, Carneiro ALFC, Costa CM, Monteiro ELO, Aristizabal LYG, et al. Association between multimorbidity, intensive care unit admission, and death in patients with COVID-19 in Brazil: a cross-section study, 2020. Sao Paulo medical journal = Revista paulista de medicina. 2022;141(3):e2022226.

38. Catalano A, Dansero L, Gilcrease W, Macciotta A, Saugo C, Manfredi L, et al. Multimorbidity and SARS-CoV-2-Related Outcomes: Analysis of a Cohort of Italian Patients. JMIR public health and surveillance. 2023;9:e41404.

39. Colnago M, Benvenuto GA, Casaca W, Negri RG, Fern, es EG, et al. Risk Factors Associated with Mortality in Hospitalized Patients with COVID-19 during the Omicron Wave in Brazil. Bioeng. 2022;9(10).

40. Covino M, Russo A, Salini S, De Matteis G, Simeoni B, Della Polla D, et al. Frailty Assessment in the Emergency Department for Risk Stratification of COVID-19 Patients Aged ≥80 Years. J Am Med Dir Assoc. 2021;22(9):1845-52.e1.

41. d'Arminio Monforte A, Tavelli A, Bai F, Tomasoni D, Falcinella C, Castoldi R, et al. The importance of patients' case-mix for the correct interpretation of the hospital fatality rate in COVID-19 disease. International Journal of Infectious Diseases. 2020;100:67-74.

42. d'Etienne JP, Alanis N, Chou E, Garrett JS, Kirby JJ, Bryant DP, et al. Validation of a simplified comorbidity evaluation predicting clinical outcomes among patients with coronavirus disease 2019 - A multicenter retrospective observation study. Am J Emerg Med. 2022;56:57-62.

43. Dandachi D, Geiger G, Montgomery MW, Karmen-Tuohy S, Golzy M, Antar AAR, et al. Characteristics, Comorbidities, and Outcomes in a Multicenter Registry of Patients With Human Immunodeficiency Virus and Coronavirus Disease 2019. Clinical infectious diseases : an official publication of the Infectious Diseases Society of America. 2021;73(7):e1964-e72.

44. Dantas ACS, Oliveira HBM, Gomes CP, Alves DL, Infante PDB, Caitité RJA, et al. Retrospective Analysis of the SARS-CoV-2 Infection Profile in COVID-19 Positive Patients in Vitoria da Conquista, Northeast Brazil. Viruses. 2022;14(11).

45. de Oliveira Lima H, da Silva LM, de Campos Vieira Abib A, Tavares LR, Santos DWCL, de Araújo ACLF, et al. Coronavirus disease-related in-hospital mortality: a cohort study in a private healthcare network in Brazil. Scientific Reports. 2022;12(1).

46. Di Fusco M, Vaghela S, Moran MM, Lin J, Atwell JE, Malhotra D, et al. COVID-19-associated hospitalizations among children less than 12 years of age in the United States. J Med Econ. 2022;25(1):334-46.

47. Díaz-Vélez C, Urrunaga-Pastor D, Romero-Cerdán A, Peña-Sánchez ER, Fernández Mogollon JL, Cossio Chafloque JD, et al. Risk factors for mortality in hospitalized patients with COVID-19 from three hospitals in Peru: A retrospective cohort study. F1000Research. 2021;10.

48. Dragano N, Dortmann O, Timm J, Mohrmann M, Wehner R, Rupprecht CJ, et al. Association of Household Deprivation, Comorbidities, and COVID-19 Hospitalization in Children in Germany, January 2020 to July 2021. JAMA network open. 2022;5(10):e2234319.

49. Elavarasi A, Raju Sagiraju HK, Garg RK, Ratre B, Sirohiya P, Gupta N, et al. Clinical features, demography, and predictors of outcomes of SARS-CoV-2 infection at a tertiary care hospital in India: A cohort study. Lung India. 2022;39(1):16-26.

50. Fagard K, Gielen E, Deschodt M, Devriendt E, Flamaing J. Risk factors for severe COVID-19 disease and death in patients aged 70 and over: a retrospective observational cohort study. Acta clinica Belgica. 2022;77(3):487-94.

51. Farrar DS, Drouin O, Moore Hepburn C, Baerg K, Chan K, Cyr C, et al. Risk factors for severe COVID-19 in hospitalized children in Canada: A national prospective study from March 2020–May 2021. Lancet Reg Health - Am. 2022;15:100337.

52. Funk T, Innocenti F, Dias JG, Nerl, er L, Melillo T, et al. Age-specific associations between underlying health conditions and hospitalisation, death and in-hospital death among confirmed COVID-19 cases: a multi-country study based on surveillance data, June to December 2020. Eurosurveillance. 2022;27(35).

53. Galang RR, Newton SM, Woodworth KR, Griffin I, Oduyebo T, Sancken CL, et al. Risk Factors for Illness Severity Among Pregnant Women With Confirmed Severe Acute Respiratory Syndrome Coronavirus 2 Infection-Surveillance for Emerging Threats to Mothers and Babies Network, 22 State, Local, and Territorial Health Departments, 29 March 2020-5 March 2021. Clin Infect Dis. 2021;73(Suppl 1):S17-S23.

54. Gani YM, Chidambaram SK, Heng BSL, Sothivadivel S, Xin LW, Aziz AA, et al. Identification of warning signs in Malaysian patients having COVID-19 infection who progress to severe form of the illness. Med J Malays. 2022;77(2):189-95.

55. Guan W-J, Liang W-H, Zhao Y, Liang H-R, Chen Z-S, Li Y-M, et al. Comorbidity and its impact on 1590 patients with COVID-19 in China: a nationwide analysis. The European respiratory journal. 2020;55(5).

56. Hardelid P, Favarato G, Wijlaars L, Fenton L, McMenamin J, Clemens T, et al. SARS-CoV-2 tests, confirmed infections and COVID-19-related hospital admissions in children and young people: birth cohort study. BMJ paediatrics open. 2022;6(1).

57. Hendler JV, do Lago PM, Muller GC, Santana JC, Piva JP, Daudt LE. Risk factors for severe COVID-19 infection in Brazilian children. Brazilian Journal of Infectious Diseases. 2021;25(6):8-.

58. Henkens MTHM, Raafs AG, Verdonschot JAJ, Linschoten M, van Smeden M, Wang P, et al. Age is the main determinant of COVID-19 related in-hospital mortality with minimal impact of pre-existing comorbidities, a retrospective cohort study. BMC geriatrics. 2022;22(1):184.

59. Hesni E, Sayad B, Khosravi Shadmani F, Najafi F, Khodarahmi R, Rahimi Z, et al. Demographics, clinical characteristics, and outcomes of 27,256 hospitalized COVID-19 patients in Kermanshah Province, Iran: a retrospective one-year cohort study. BMC Infectious Diseases. 2022;22(1).

60. Ho FK, Petermann-Rocha F, Gray SR, Jani BD, Katikireddi SV, Niedzwiedz CL, et al. Is older age associated with COVID-19 mortality in the absence of other risk factors? General population cohort study of 470,034 participants. PloS one. 2020;15(11):e0241824.

61. Houvèssou GM, Vargas PCG, Jacques N, Leventhal DGP. Risk factors for intensive care unit admission and death from COVID-19 in fully vaccinated patients hospitalized for severe COVID-19, Brazil, 2021–2022. Rev Panam Salud Publica Pan Am J Public Health. 2022;46.

62. Impouma B, Carr ALJ, Spina A, Mboussou F, Ogundiran O, Moussana F, et al. Time to death and risk factors associated with mortality among COVID-19 cases in countries within the WHO African region in the early stages of the COVID-19 pandemic. Epidemiology and Infection. 2022.

63. Islam MS, Bhowmick DK, Parveen M, Kamal MM, Akhtaruzzaman AKM. Case fatality rate and survival functions of severe COVID-19 patients in intensive care unit of Bangabandhu Sheikh Mujib Medical University in Bangladesh: An observational study. Anaesth Pain Intensive Care. 2021;25(4):443-9.

64. Ismail M, Joudeh A, Al-Dahshan A, Alsaadi MM, Al Abdulla S, Selim NAA. Spectrum of COVID-19 clinical characteristics among patients presenting to the primary healthcare in Qatar during the

early stages of the pandemic: A retrospective multicentre cross-sectional study. BMJ Open. 2021;11(12).

65. Izzy S, Tahir Z, Cote DJ, Al Jarrah A, Roberts MB, Turbett S, et al. Characteristics and outcomes of latinx patients with COVID-19 in comparison with other ethnic and racial groups. Open Forum Infectious Diseases. 2020;7(10):1-11.

66. Jachymek M, Cader A, Ptak M, Witkiewicz W, Szymanski AG, Kotfis K, et al. The Value of Clinical Frailty Scale (CFS) as a Prognostic Tool in Predicting Mortality in COVID-19-A Retrospective Cohort Study. International journal of environmental research and public health. 2022;19(3).

67. Jesmani Y, Bozorgomid A, Shadmani FK, Dehbani A, Sayad B. Demographic and clinical characteristics and outcomes of COVID-19 patients admitted to a university hospital in the west of Iran: a retrospective study in the third wave. Vacunas. 2023.

68. Kammar-García A, de Vidal-Mayo JJ, Vera-Zertuche JM, Lazcano-Hernández M, Vera-López O, Segura-Badilla O, et al. Impact of comorbidities in Mexican SARS-CoV-2-positive patients: A retrospective analysis in a national cohort. Rev Invest Clin. 2020;72(3):151-8.

69. Khalifa AM, Nouh FA, Elshaari FA. Clinical characteristics and outcomes among patients with COVID-19. Saudi Medical Journal. 2022;43(9):1013-9.

70. Kofahi HM, Swedan SF, Khabour OF, Nimer RM. Predictors of COVID-19 severity and hospitalization: A survey-based study from Jordan. Inform Med Unlocked. 2022;31.

71. Kokoszka-Bargieł I, Cyprys P, Madeja P, Rutkowska K, Wajda-Pokrontka M, Madowicz J, et al. Factors influencing death in COVID-19 patients treated in the ICU: a single-centre, cross-sectional study. Anaesthesiology Intensive Therapy. 2022;54(2):132-40.

72. Koyyada R, Nagalla B, Tummala A, Singh AD, Patnam S, Barigala R, et al. Prevalence and Impact of Preexisting Comorbidities on Overall Clinical Outcomes of Hospitalized COVID-19 Patients. BioMed research international. 2022;2022:2349890.

73. Krüger S, Dun L, Joseph JA, van Hoving N, Phillips L. Comorbidities associated with COVID-19 mortality: A retrospective study in an intermediate care facility in Cape Town, South Africa. Southern African Journal of Public Health. 2022;5(3):86-92.

74. Kuhn KG, Khadka K, Adesigbin K, Altidort B, Boyina K, Withers E, et al. Characterization of persons with reported SARS-CoV-2 infection in the Oklahoma City tri-county area: Evidence from the first 12 months of transmission. American Journal of Infection Control. 2022.

75. Kumar H, Dixit S, Gupta N, Gupta P, ey MK, Shakya S, et al. Impact of Co-morbidities on outcome of COVID-19 patients: An observational study among patients admitted to intensive care unit. Journal of Clinical and Diagnostic Research. 2021;15(7):48-51.

76. Lam B, Stepanova M, Venkatesan C, Garcia I, Reyes M, Mannan A, et al. Outcomes of hospitalized patients with COVID-19 during the course of the pandemic in a fully integrated health system. PloS one. 2022;17(2):e0263417.

77. Larsson E, Brattström O, Agvald-Öhman C, Grip J, Campoccia Jalde F, Strålin K, et al. Characteristics and outcomes of patients with COVID-19 admitted to ICU in a tertiary hospital in Stockholm, Sweden. Acta Anaesthesiologica Scandinavica. 2020.

78. Levy Y, Turjeman A, Cooper L, Kagansky N, Nagulevich T, Snir T, et al. Frail Older Adults with Presymptomatic SARS-CoV-2 Infection: Clinical Course and Prognosis. Gerontology. 2022;68(12):1393-401.

79. Lota-Salvado R, Padua JR, Agrupis KA, Malijan GM, Sayo AR, Suzuki S, et al. Epidemiological and clinical characteristics of children with confirmed COVID-19 infection in a tertiary referral hospital in Manila, Philippines. Trop Med Health. 2023;51(1).

80. Marengoni A, Zucchelli A, Vetrano DL, Armellini A, Botteri E, Nicosia F, et al. Beyond Chronological Age: Frailty and Multimorbidity Predict In-Hospital Mortality in Patients With Coronavirus Disease 2019. The journals of gerontology Series A, Biological sciences and medical sciences. 2021;76(3):e38-e45.

81. Marin-Gomez FX, Mendioroz-Pena J, Mayer M-A, Mendez-Boo L, Mora N, Hermosilla E, et al. Comparing the Clinical Characteristics and Mortality of Residential and Non-Residential Older People with COVID-19: Retrospective Observational Study. International journal of environmental research and public health. 2022;19(1).

82. Marti-Pastor A, Moreno-Perez O, Lobato-Martinez E, Valero-Sempere F, Amo-Lozano A, Martinez-Garcia M-A, et al. Association between Clinical Frailty Scale (CFS) and clinical presentation and outcomes in older inpatients with COVID-19. BMC geriatrics. 2023;23(1):1.

83. Mertens E, Serrien B, romme M, Peñalvo JL, the Belgian Collaborative Group on C-HS. Predicting COVID-19 progression in hospitalized patients in Belgium from a multi-state model. Frontiers in Medicine. 2022;9.

84. Mi J, Zhong W, Huang C, Zhang W, Tan L, Ding L. Gender, age and comorbidities as the main prognostic factors in patients with COVID-19 pneumonia. Am J Transl Res. 2020;12(10):6537-48.

85. Mohammadifard N, Haghighatdoost F, Nasirian M, Zakeri P, Heidari K, Haghjooy Javanmard S, et al. Sex Differences in the Relation between Comorbidities and Prognosis in Hospitalized Patients with COVID-19. Interdiscip Perspect Infect Dis. 2022;2022.

86. Monari C, Pisaturo M, Maggi P, Macera M, Di Caprio G, Pisapia R, et al. Early predictors of clinical deterioration in a cohort of outpatients with COVID-19 in southern Italy: A multicenter observational study. Journal of Medical Virology. 2022;94(11):5336-44.

87. Ngere P, Onsongo J, Langat D, Nzioka E, Mudachi F, Kadivane S, et al. Characterization of COVID-19 cases in the early phase (March to July 2020) of the pandemic in Kenya. Journal of global health. 2022;12:15001.

88. Oliveira EA, Oliveira MCL, Silva ACSE, Colosimo EA, Mak RH, Vasconcelos MA, et al. Clinical Outcomes of Omicron Variant (B.1.1.529) Infection in Children and Adolescents Hospitalized with COVID-19 in Brazil with Observational Data on the Efficacy of the Vaccines in Adolescents. Pediatr Infect Dis J. 2023;42(3):218-25.

89. Pinzon RT, Veronica V. Medical comorbidities as predictors of COVID-19 short-term mortality: A historical cohort study in Indonesia. Tzu Chi Med J. 2023;35(1):53-7.

90. Polverino F, Stern DA, Ruocco G, Balestro E, Bassetti M, Candelli M, et al. Comorbidities, cardiovascular therapies, and COVID-19 mortality: A nationwide, italian observational study (ItaliCO). Frontiers in Cardiovascular Medicine. 2020;7.

91. Quenzer FC, Coyne CJ, Ferran K, Williams A, Lafree AT, Kajitani S, et al. ICU Admission Risk Factors for Latinx COVID-19 Patients at a U.S.-Mexico Border Hospital. Journal of Racial and Ethnic Health Disparities. 2022.

92. Rainer L, Bachner F, Eglau K, Ostermann H, Siebert U, Zuba M. Comorbidities and COVID-19 hospitalization, ICU admission and hospital mortality in Austria : A retrospective cohort study. Wiener klinische Wochenschrift. 2022;134(23-24):856-67.

93. Rana R, Ranjan V, Kumar N, Chugh P, Khillan K, Gogia A, et al. Association of underlying comorbidities and progression of COVID-19 infection amongst 2586 patients hospitalised in the National Capital Region of India: a retrospective cohort study. Mol Cell Biochem. 2023;478(1):149-60.

94. Rando E, Oliva A, Cancelli F, D'agostino C, Savelloni G, Ciardi MR, et al. Clinical characteristics and risk factors for mortality in COVID-19 patients during the first wave of the COVID-19 pandemic in Rome, Italy: a single-center retrospective study. Infez Med. 2023;31(1):49-54.

95. Rossi PG, Marino M, Formisano D, Venturelli F, Vicentini M, Grilli R, et al. Characteristics and outcomes of a cohort of COVID-19 patients in the Province of Reggio Emilia, Italy. PLoS ONE. 2020;15(8 August).

96. Semenzato L, Botton J, Drouin J, Cuenot F, Dray-Spira R, Weill A, et al. Chronic diseases, health conditions and risk of COVID-19-related hospitalization and in-hospital mortality during the first wave of the epidemic in France: A cohort study of 66 million people. Pharmacoepidemiology and Drug Safety. 2021;30:21-2.

97. Siddiqi Z, Fatima J, Bhatt D, Shukla V, Malik M, Ashfaq A, et al. Prevalence of Comorbidities in Survivors and Non-Survivors of Severe COVID-19 at a Dedicated COVID Care Centre. The Journal of the Association of Physicians of India. 2022;70(1):11-2.

98. Simoes E Silva AC, Vasconcelos MA, Colosimo EA, Mendonca ACQ, Martelli-Junior H, Silva LR, et al. Outcomes and risk factors of death among hospitalized children and adolescents with obesity and COVID-19 in Brazil: An analysis of a nationwide database. Pediatric obesity. 2022;17(9):e12920.

99. Siqueira TS, De Souza EKG, Martins-Filho PR, Silva JRS, Gurgel RQ, Cuevas LE, et al. Clinical characteristics and risk factors for maternal deaths due to COVID-19 in Brazil: a nationwide population-based cohort study. Journal of Travel Medicine. 2022;29(3).

100. Skarbinski J, Wood MS, Chervo TC, Schapiro JM, Elkin EP, Valice E, et al. Risk of severe clinical outcomes among persons with SARS-CoV-2 infection with differing levels of vaccination during widespread Omicron (B.1.1.529) and Delta (B.1.617.2) variant circulation in Northern California: A retrospective cohort study. Lancet Reg Health - Am. 2022;12.

101. Smith JP, Kressel AB, Grout R, W. a, Weaver B, Cheatham M, et al. Poverty, Comorbidity, and Ethnicity: COVID-19 Outcomes in a Safety Net Health System. Ethnicity & disease. 2022;32(2):113-22.

102. Solanki G, Wilkinson T, Bansal S, Shiba J, a S, Doherty T. COVID-19 hospitalization and mortality and hospitalization-related utilization and expenditure: Analysis of a South African private health insured population. PLoS ONE. 2022;17(5).

103. Subramaniam A, Shekar K, Anstey C, Tiruvoipati R, Pilcher D. Impact of frailty on clinical outcomes in patients with and without COVID-19 pneumonitis admitted to intensive care units in Australia and New Zealand: a retrospective registry data analysis. Crit Care. 2022;26(1).

104. Sundaram SS, Melquist S, Kalgotra P, Srinivasan S, Parasa S, Desai M, et al. Impact of age, sex, race, and regionality on major clinical outcomes of COVID-19 in hospitalized patients in the United States. BMC infectious diseases. 2022;22(1):659.

105. Surendra H, Praptiningsih CY, Ersanti AM, Rahmat M, Noviyanti W, Harman AD, et al. Clinical characteristics and factors associated with COVID-19-related mortality and hospital admission during the first two epidemic waves in 5 rural provinces in Indonesia: A retrospective cohort study. PLoS ONE. 2023;18(3 March).

106. Tam EMYY, Kwan YK, Ng YY, Yam PW. Clinical course and mortality in older patients with COVID-19: a cluster-based study in Hong Kong. Hong Kong medical journal = Xianggang yi xue za zhi. 2022;28(3):215-22.

107. Toofan F, Hosseini SM, Alimohammadzadeh K, Jafari M, Bahadori M. Impact of comorbidities on mortality in hospitalized patients with COVID-19: An experience from Iran. Journal of Education and Health Promotion. 2021;10(1).

108. Traiber C, Bueno FU, Braun Filho LR, Eckert GU, Azambuja MA, Gras GS. Pediatric patients with COVID-19 admitted to a PICU in Southern Brazil, excluding MIS-C. Acta Colomb Cuid Intensiv. 2022;22:S46-S54.

109. Wiley Z, Kulshreshtha A, Li D, Kubes J, iah S, Leung S, et al. Clinical characteristics and social determinants of health associated with 30-day hospital readmissions of patients with COVID-19. Journal of Investigative Medicine. 2022;70(6):1406-15.

110. Zhang C, Lu W, Wang W, Yi J, Wu G, Zhang X, et al. Clinical characteristics of severe and critical coronavirus disease and assessment of risk factors for progression: a retrospective cohort study. Acta Medica Mediterranea. 2022;38(1):395-403.

111. Zhang Y, Luo W, Li Q, Wang X, Chen J, Song Q, et al. Risk Factors for Death among the First 80 543 Coronavirus Disease 2019 (COVID-19) Cases in China: Relationships between Age, Underlying Disease, Case Severity, and Region. Clinical Infectious Diseases. 2022;74(4):630-8.
